# Functional cRGD-Conjugated Polymer Prodrug for Targeted Drug Delivery to Liver Cancer Cells

**DOI:** 10.1021/acsomega.2c02683

**Published:** 2022-06-07

**Authors:** Ru Zhou, Mingzu Zhang, Jinlin He, Jian Liu, Xingwei Sun, Peihong Ni

**Affiliations:** † College of Chemistry, Chemical Engineering and Materials Science, State and Local Joint Engineering Laboratory for Novel Functional Polymeric Materials, Jiangsu Key Laboratory of Advanced Functional Polymer Design and Application, Suzhou Key Laboratory of Macromolecular Design and Precision Synthesis, Soochow University, Suzhou 215123, P. R. China; ‡ Institute of Functional Nano and Soft Materials (FUNSOM), Soochow University, Suzhou 215123, P. R. China; § Intervention Department, The Second Affiliated Hospital of Soochow University, Suzhou 215004, P. R. China

## Abstract

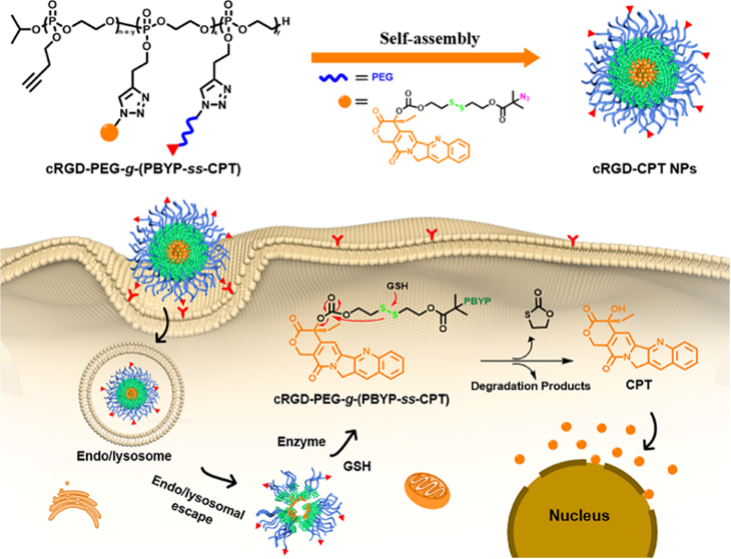

To overcome the limitation of conventional nanodrugs in tumor targeting efficiency, coupling targeting ligands to polymeric nanoparticles can enhance the specific binding of nanodrugs to tumors. Cyclo(Arg-Gly-Asp-d-Phe-Lys) (abbreviated as c(RGDfK)) peptide has been widely adopted due to its high affinity to the tumor marker α_v_β_3_ integrin receptor. In this study, we develop a cRGD peptide-conjugated camptothecin (CPT) prodrug, which enables self-assembly of nanoparticles for precise targeting and enrichment in tumor tissue. We first synthesized a camptothecin derivative (CPT-*ss*-N_3_) with a reduction-sensitive bond and simultaneously modified PEG to obtain cRGD-PEG-N_3_. After ring-opening polymerization of the 2-(but-3-yn-1-yolxy)-2-oxo-1,3,2-dioxaphospholane (BYP), an amphiphilic polymeric prodrug, referred to as cRGD-PEG-*g*-(PBYP-*ss*-CPT), was obtained via copper-catalyzed azide–alkyne cycloaddition (CuAAC) reaction. The self-assembly in buffer solution of the cRGD-functional prodrug was studied through DLS and TEM. The *in vitro* drug release behavior of cRGD-PEG-*g*-(PBYP-*ss*-CPT) nanoparticles was investigated. The results show that the nanoparticles are reduction-responsive and the bonded CPT can be released. Endocytosis and MTT assays demonstrate that the cRGD-conjugated prodrug has better affinity for tumor cells, accumulates more intracellularly, and is therefore, more effective. The *in vivo* drug metabolism studies show that nanoparticles greatly prolong the retention time in circulation. By monitoring drug distribution in tumor and in various tissues, we find that free CPT can be rapidly metabolized, resulting in low accumulation in all tissues. However, cRGD-PEG-*g*-(PBYP-*ss*-CPT) nanoparticles accumulate in tumor tissues in higher amounts than PEG-*g*-(PBYP-*ss*-CPT) nanoparticles, except for the inevitable capture by the liver. This indicates that the nanomedicine with cRGD has a certain targeting property, which can improve drug delivery efficiency.

## Introduction

Globally, despite rapid advances in medical technology, cancer remains the second leading cause of death according to the statistics conducted by the American Cancer Society in 2021,^1^ and the high cost of treatment leaves many patients without effective treatment.^2^ Liver cancer is considered the fifth most common cancer, with a 5-year survival rate of only 20% from 2010 to 2016.^1^ To reduce costs and minimize patient suffering, various tools have been developed for cancer treatment, and multifunctional nanomaterials for tumor treatment and imaging technologies have been widely noticed.^3^ Although the accumulation of nanomedicines in tumors can be high above traditional small-molecule drugs, it still amounts to only 5–10% of the total drug injected.^4,5^ Therefore, there are many challenges in developing more targeted affinity drug delivery methods and reducing biotoxicity on normal tissues.

It has been shown that when the nanoparticle shells are combined with targeting molecules, the active nanoparticles can enhance affinity and binding ability to tumor cells via receptor-mediated endocytosis.^6,7^ Functional ligand-conjugated polymers achieve higher efficiency in drug delivery due to the selective recognition of specific markers on tumor cell membranes, including epidermal growth factor receptors, integrins, transferrin receptors, integrins, etc.^8−11^ In recent years, the field of α_v_β_3_ integrin-mediated bioactive tumor targeting has been explored extensively. α_v_β_3_ integrins are overexpressed on a variety of tumor cells, including hepatocellular carcinoma cells,^12^ breast cancer cells,^13^ and lung cancer cells.^14^ Also, α_v_β_3_ integrins are associated with tumor growth progression and metastasis.^15,16^ Cyclic RGDfK (cRGD) has a high affinity for α_v_β_3_ integrins, which makes it one of the ideal ligands for use in targeted therapies. Among the target molecules reported in the literature so far, cRGD is able to participate in a variety of chemical reactions without inactivation, and has the advantage of easy endocytosis due to a small molecular weight. Meanwhile, on account of the “chelating effect”, integrin α_v_β_3_ has high affinity and selectivity with cyclic RGD peptides.^17^ Targeting tumor vessels or cells by cRGD modification of nanocarriers for delivery of chemotherapeutic agents and imaging agents have been intensively explored by researchers.^18−20^

The most common nanocarriers used in clinical practice are liposomes, polymers, iron oxide nanoparticles, carbon nanotubes, gold nanoparticles, etc.^21,22^ Among them, polymeric carriers are structurally diverse. Both natural and synthetic polymers can be modified to obtain the desired properties.^23^ Since the 1960s, researchers have worked in the field of polymeric controlled release of drugs and have developed new approaches to synthetic methods and bioconjugation techniques.^24,25^ The launch of the first polymer-based nanomedicine, Genexol-PM^26^ [poly(ethylene glycol)-polylactic acid (PEG-PLA) micellarized paclitaxel], marked the beginning of the entry of polymeric nanocarriers into clinical applications. Drug-loaded polymers should be biocompatible and metabolizable in the body, for instance, polyphosphoesters (PPEs),^27^ poly(ethylene glycol) (PEG),^28^ polypeptides,^29^ etc. Stimuli-responsive drug carriers can be skillfully designed for endogenous stimuli (pH,^30^ redox^31^) and exogenous stimuli (magnetic field,^32^ laser irradiation^33^) of the tumor microenvironment.

Unlike normal vasculature, tumor tissue has chaotic and disorganized vasculature stems, which are determining elements in the ability of nanoparticles to penetrate into the tumor.^34^ Many previous works have been devoted to the study of modified polymers with stimuli-responsive groups to construct polymeric prodrugs for efficient and controlled drug release in specific environments. For example, tumor cells possess a reducing microenvironment in which the concentration of glutathione (GSH) is as high as 2–10 mM, 100–1000 times higher than normal tissues.^35^ Therefore, it is considered as an ideal and prevalent endogenous stimulus to rapidly disrupt the sensitive bonds of intracellular nanocarriers, resulting in efficient intracellular drug release.^36,37^ To address this feature, in our previous works, we reported the [PEEP-*b*-PBYP-Se]_2_ prodrug^38^ and CPT-*ss*-poly(BYP_-*hyd*-DOX_-*co*-EEP) prodrug,^39^ respectively, in which both the introduced diselenium and disulfide bonds can respond rapidly in the reductive environment.

**Scheme 1:**
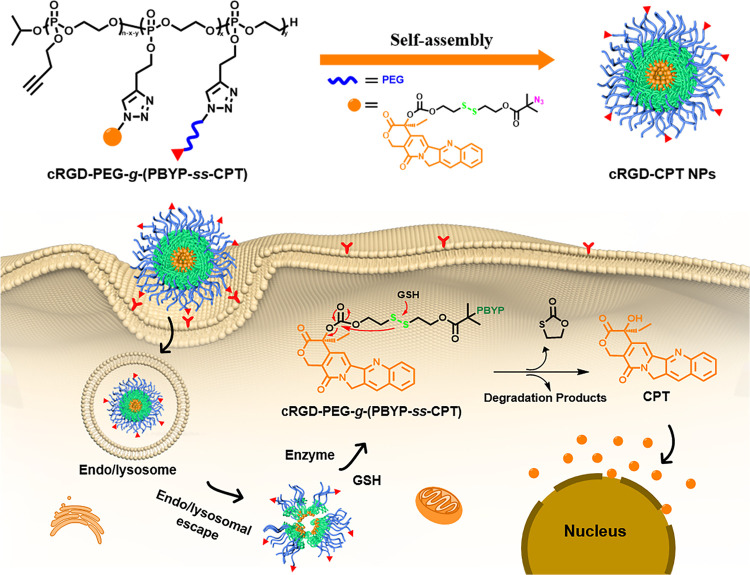
Schematic Diagram of Endocytosis by Self-Assembled cRGD-PEG-*g*-(PBYP-*ss*-CPT) Nanoparticles and Triggering of CPT Release in the Reducing Environment of Cancer Cells

In this research, we aimed to increase the accumulation and targeted release of camptothecin at tumor sites, thereby reducing systemic toxicities. As shown in Scheme 1, we chose cRGD as the targeting molecule, polyphosphoester as the drug carrier, and subsequently grafted poly(ethylene glycol) to construct stimuli-responsive polymeric prodrugs for precise delivery of antitumor drugs. In buffer solution with pH 7.4, polymer prodrug cRGD-PEG-*g*-(PBYP-*ss*-CPT) can self-assemble into nanoparticles. The cRGD-prodrug has the following advantages: (1) good biocompatibility and biodegradability using polyphosphoester as the drug carrier backbone; (2) prolonged drug circulation time and low renal clearance due to reduced non-specific interactions of PEG *in vivo*; (3) stable encapsulation of hydrophobic drugs by the disulfide bond linkage without drug leakage under physiological conditions; and (4) increased drug accumulation in the tumor tissues and selective drug delivery to the therapeutic target.

## Experimental Section

The experimental section contains two parts, chemical synthesis methods and test characterization methods, details of which are in the Supporting Information. The chemical synthesis involves the functionalized modification of CPT and cRGD, the preparation of PBYP by ring-opening polymerization, and the preparation of cRGD-PEG-*g*-(PBYP-*ss*-CPT) by one-pot click reaction. The section on test characterization methods covers the detailed steps for *in vitro* and *in vivo* effect evaluation of polymeric prodrugs.

## Results and Discussion

### Synthesis of the cRGD-prodrug Conjugate

**Scheme 2:**
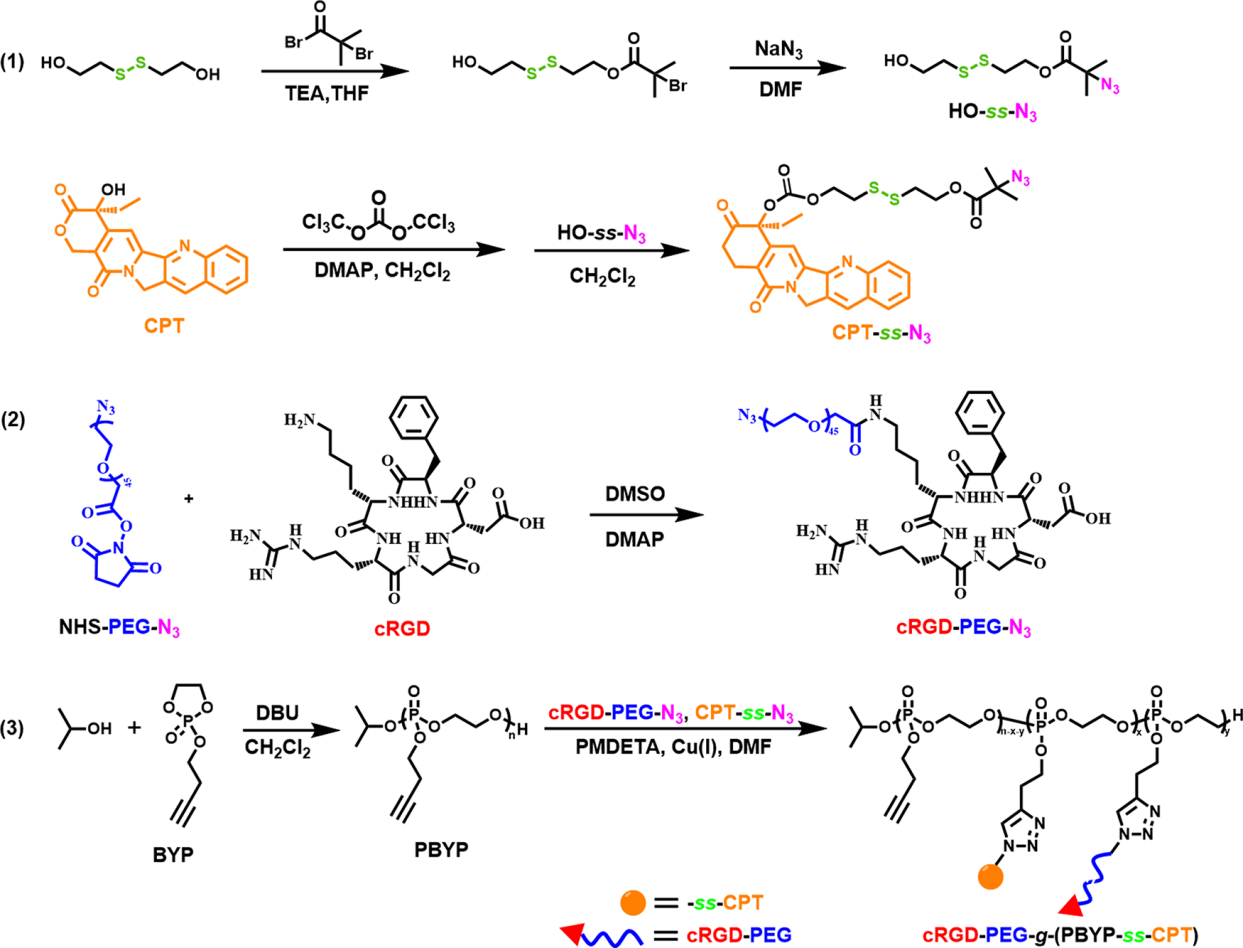
Synthetic Routes to cRGD-PEG-*g*-(PBYP-*ss*-CPT)

We prepared polyphosphoester-based cRGD-prodrug conjugates through three steps, as indicated in Scheme 2. First, the CPT derivative (CPT-*ss*-N_3_) with a disulfide bond was synthesized and functionalized cRGD-PEG-N_3_ was prepared. Subsequently, we obtained an amphiphilic cRGD-PEG-*g*-(PBYP-*ss*-CPT) through ring-opening polymerization, and following one-pot CuAAC reaction between CPT-*ss*-N_3_, cRGD-PEG-N_3_, and PBYP. Both cRGD-PEG-N_3_ and CPT-*ss*-N_3_ were grafted onto the side groups of the PBYP backbone. To simplify the name, we abbreviate the polymer prodrug as cRGD-PEG-*g*-(PBYP-*ss*-CPT).

Multiple characterization methods confirmed the successful synthesis of the polymer prodrug. Figure S1 in the Supporting Information shows the ^1^H NMR spectra of HO-*ss*-Br, HO-*ss*-N_3_, and CPT-*ss*-N_3_. The detailed peaks correspond to the protons of the three compounds and almost no impurity peaks are observed, validating that the chemical structures of the three compounds are correct.

**Figure 1:**
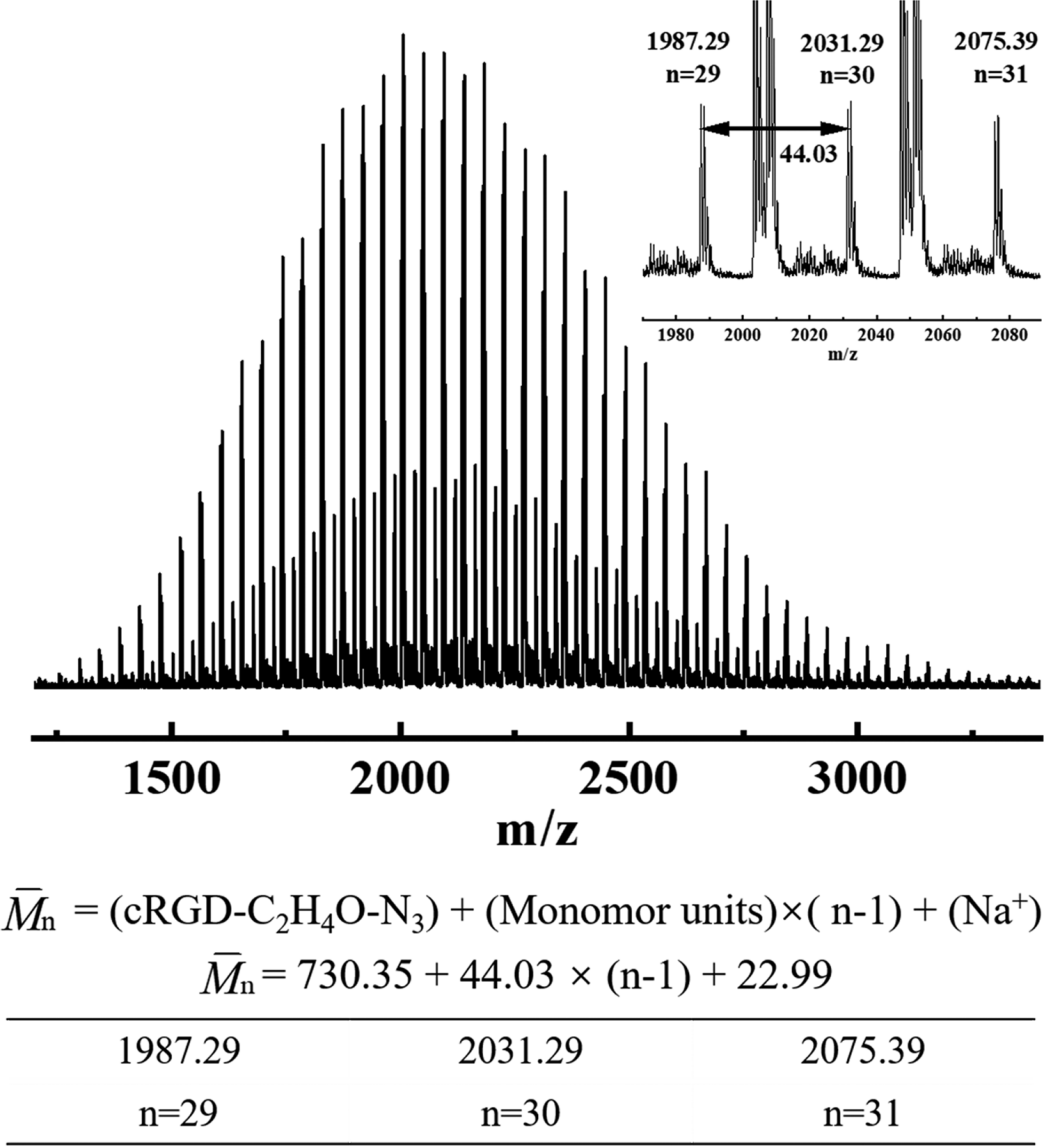
MALDI-TOF MS spectrum of cRGD-PEG-N_3_.

To verify the results of the modification of PEG with cRGD, the structure of cRGD-PEG-N_3_ was tested using ^1^H NMR and MALDI-TOF MS. In Figure S2, the characteristic peaks at δ 7.24–7.37 ppm attributed to phenyl protons of cRGD (peaks 10, 11, 12) can be detected, indicating that cRGD-PEG-N_3_ has been synthesized successfully. The degree of functionalization is 72%, which is calculated by eq 1
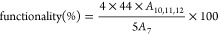
1where *A*_10,11,12_ and *A*_7_ are the corresponding integral areas in Figure S2, respectively, and 44 represents the molar mass of each structural unit in PEG. To further prove that cRGD and NHS-PEG-N_3_ are chemically bonded rather than simply mixed, we used MALDI-TOF MS to analyze the reaction products of cRGD and NHS-PEG-N_3_. According to Figure 1, after molecular weight calculations of cRGD-PEG-N_3_ with various repeating units, we can confirm that the product is cRGD-PEG-N_3_ and a small amount of unreacted NHS-PEG-N_3_.

**Figure 2:**
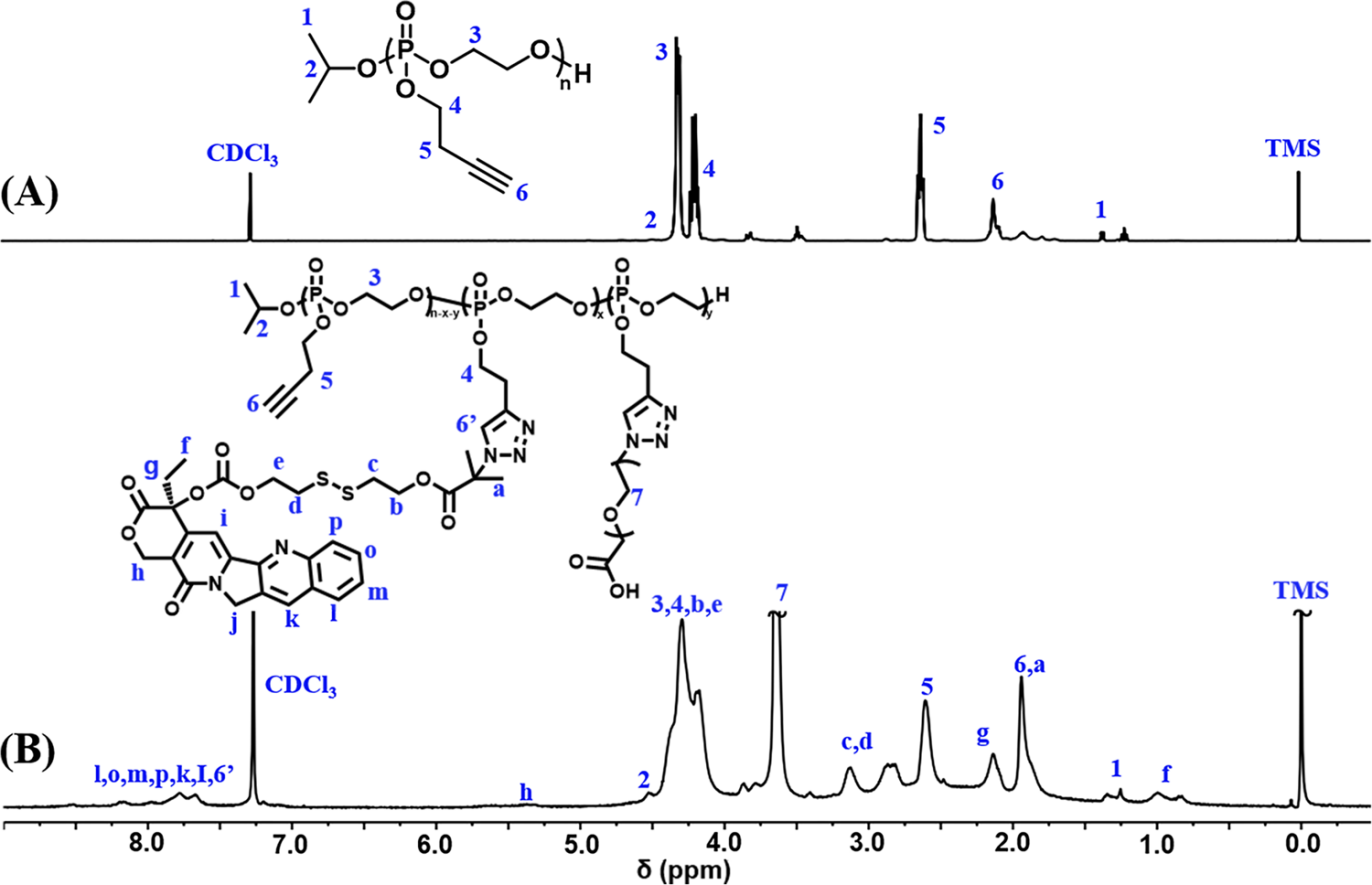
^1^H NMR spectra of (A) PBYP_56_ and (B) PEG-*g*-(PBYP_56_-*ss*-CPT) (solvent: CDCl_3_).

In addition, we used ^1^H NMR and GPC to verify PBYP and the amphiphilic graft copolymer PEG-*g*-(PBYP-*ss*-CPT), respectively. Figure 2 displays the ^1^H NMR spectra of PBYP and PEG-*g*-(PBYP-*ss*-CPT). We can first calculate the degree of polymerization (*n*) of PBYP by eq 2, and then calculate the relative molecular weight of PBYP by the eq 3
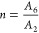
2
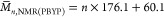
3where *A*_6_ is the integrated area of the alkynyl proton (−CH_2_C≡C***H***) in PBYP and *A*_2_ is the integral area of the proton (−C***H***(CH_3_)_2_) in isopropanol. 176.1 represents the molar mass of each structural unit and 60.1 is the molar mass of methine in isopropanol. According to Figure 2B, a new chemical shift at δ 3.65 ppm (peak 7, −C***H***_2_C***H***_2_O-) can be attributed to PEG. In addition, the chemical shift signals at δ 7.53–8.41 ppm are attributed to the protons of CPT and the triazole ring. The appearance of new proton peaks at the chemical shifts confirms that CPT-*ss*-N_3_ and cRGD-PEG-N_3_ have performed successful click reactions with the alkynyl groups of PBYP block.

The molecular weights (*M̅*_n_) and dispersity (*Đ*) of PBYP and PEG-*g*-(PBYP-*ss*-CPT) are listed in Table 1. In subsequent experiments, we selected the PEG-*g*-(PBYP_56_-*ss*-CPT) (*M̅*_n_ = 17 300 g mol^–1^) sample for *in vivo* and *in vitro* studies. Furthermore, Figure S3 shows the GPC curves, among them, the PEG-*g*-(PBYP-*ss*-CPT) curve had an increased efflux time in comparison to PBYP, indicating an increase in *M̅*_n_ and achievement of the graft copolymer.

**Table 1:** Molecular Weights and Dispersity of PBYP and PEG-*g*-(PBYP-*ss*-CPT)

sample	*M̅*_n_ (g mol^–1^)^a^	*M̅*_w_ (g mol^–1^)^a^	*Đ*^a^
PBYP_50_	8800	11 000	1.24
PBYP_56_	12 200	19 000	1.40
PEG-*g*-(PBYP_50_-*ss*-CPT)	11 600	15 700	1.35
PEG-*g*-(PBYP_56_-*ss*-CPT)	17 300	28 400	1.64
PEG-*g*-(PBYP_56_-*ss*-CPT)	14 400	27 400	1.90

a Measured by GPC. (eluting solvent: DMF; standard: polystyrene). The subscript PBYP indicates the degree of polymerization, which is calculated from eqs (1,2).

To demonstrate that CPT is chemically bonded to PBYP, we used HPLC to measure it. Figure S4A shows the HPLC outflow curves, in which CPT-*ss*-N_3_ eluted at 6.48 min, whereas PEG-*g*-(PBYP-*ss*-CPT) had an efflux time of 1.75 min, indicating that the product had been purified and there was no residual CPT-*ss*-N_3_. In Figure S4B, UV–vis spectra show that the copolymer carrier did not have absorption peaks. In contrast, the absorption peaks of both free CPT and the polymer prodrug are at 365 nm, demonstrating that the prodrug PEG-*g*-(PBYP-*ss*-CPT) was successfully prepared. Meanwhile, the CPT content of different prodrugs was also measured by UV–vis spectroscopy and listed in Table 2. In subsequent experiments, we selected the cRGD-PEG-*g*-(PBYP_56_-*ss*-CPT) (*C*_CPT_ = 15.43%) sample for *in vivo* and *in vitro* studies.

**Table 2:** CPT Loading Capacity of the Polymeric Prodrug

sample	feed molar ratio of –C≡CH: −N_3_	loading capacity (wt %)^a^
PEG-*g*-(PBYP_56_-*ss*-CPT)	5:1	8.46
PEG-*g*-(PBYP_56_-*ss*-CPT)	5:1	6.77
cRGD-PEG-*g*-(PBYP_56_-*ss*-CPT)	5:1	16.22
cRGD-PEG-*g*-(PBYP_56_-*ss*-CPT)	5:1	15.43

a Calculated by *C*_CPT_ (wt %) = (*C*_test_/*C*_sample_) × 100, where *C*_sample_ is the concentration of the prodrug and *C*_test_ is UV–vis detected concentration of CPT contained therein.

### Enzyme Degradation of PBYP

**Figure 3:**
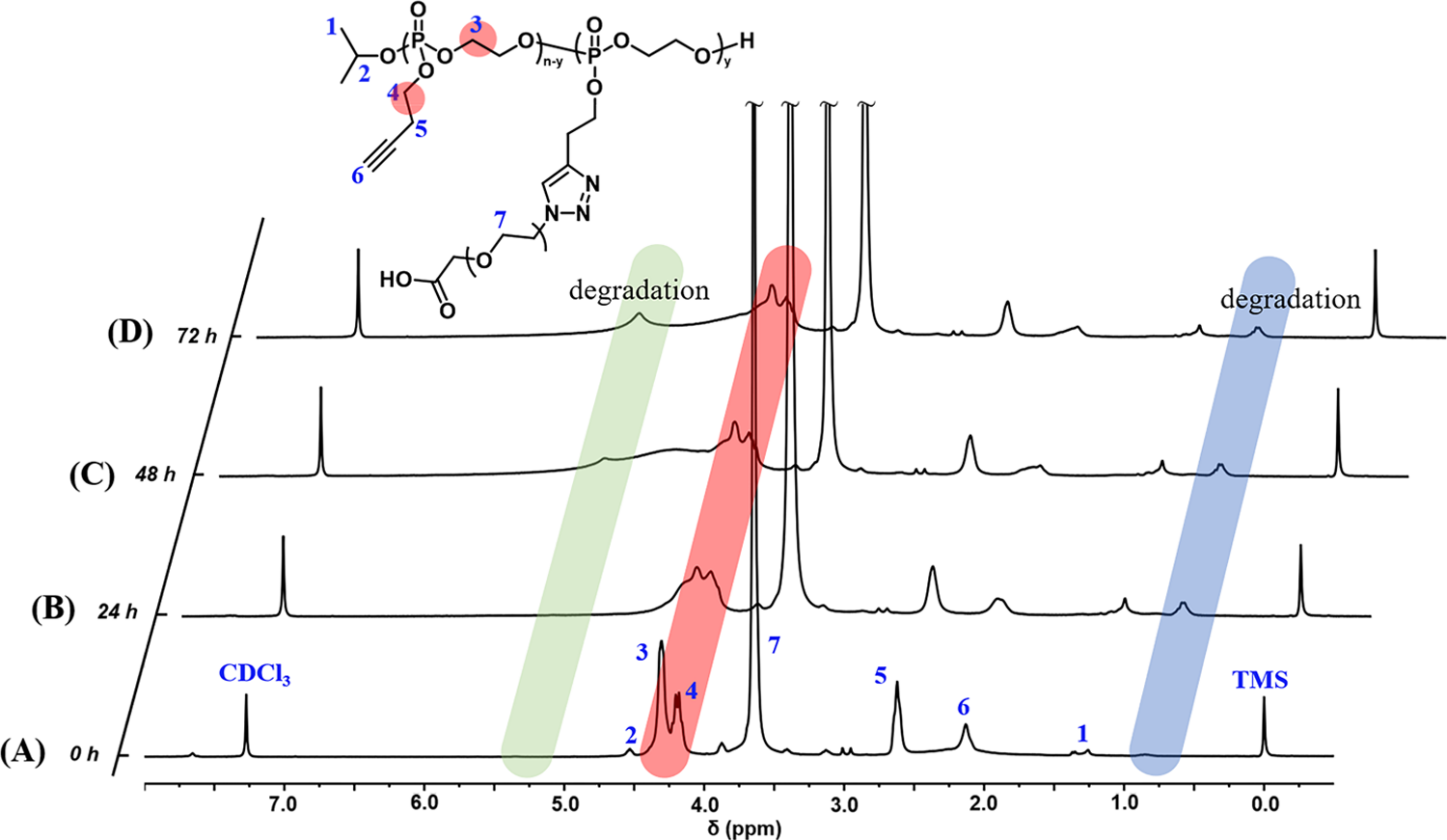
^1^H NMR spectra of (A) PEG-*g*-PBYP_56_, and its degradation products at incubation times of (B) 24 h, (C) 48 h, and (D) 72 h, respectively (solvent: CDCl_3_).

As a widely used biomedical material, polyphosphoesters have good degradability in the presence of phosphodiesterase (PDE I). The degradability can be verified by ^1^H NMR of degradation products, as shown in Figure 3. After incubation in buffer solution containing PDE I, the peaks at δ 4.32 and δ 2.36 ppm belonging to the protons of the polyphosphoester segment gradually weakened with time. Meanwhile, new peaks were observed at δ 5.25 and δ 0.86 ppm, indicating that PEG-*g*-PBYP_56_ underwent degradation and generated new degradation products. As a reference, the Wooly group and the Wurm group also used ^31^P NMR to confirm the biodegradability of PBYP in their reported literature on BYP copolymers.^40−42^

### Self-Assembly Properties of Amphiphilic Prodrugs

While the polymer concentration is high above the critical aggregation concentration (CAC), PBYP and CPT will be surrounded by PEG under hydrophilic and hydrophobic forces in aqueous solution, thus forming prodrug micelles. The micellization behavior was studied by the pyrene fluorescence probe method. The CAC value (54 mg L^–1^) was obtained after linear fitting and calculation as shown in Figure S5.

**Figure 4:**
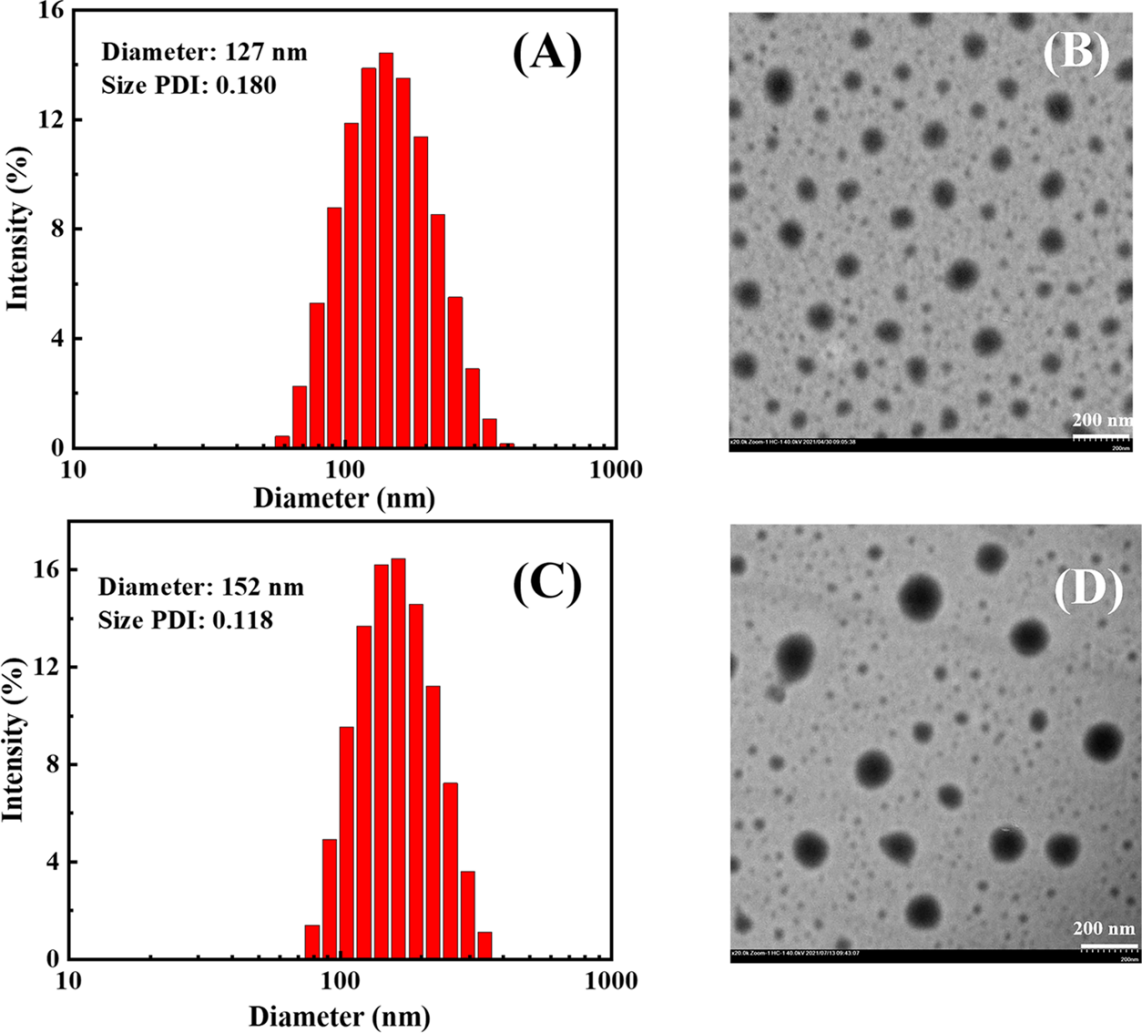
Particle size distribution histograms: (A) PEG-*g*-(PBYP-*ss*-CPT) prodrug NPs, (C) cRGD-CPT NPs and corresponding TEM images (B) and (D) (concentration: 1 mg mL^–1^, scale bar = 200 nm).

The particle size and PDI are significant parameters for nanoparticles to pass the tumor vascular barrier. Dynamic light scattering (DLS) and transmission electron microscopy (TEM) were used to evaluate the self-assembly effect of PEG-*g*-(PBYP-*ss*-CPT) and cRGD-PEG-*g*-(PBYP-*ss*-CPT), as shown in Figure 4. The average particle size (*D̅_z_*) of PEG-*g*-(PBYP-*ss*-CPT) NPs was 127 nm and the PDI was 0.180 measured by DLS, which was consistent with the TEM test result. As shown in Figure 4B, most of the nanoparticles formed from PEG-*g*-(PBYP-*ss*-CPT) are relatively uniform, and there are still some smaller particles. This may be due to the fact that the amphiphilic polymer prodrugs have a certain molecular weight and cannot form micelles with completely uniform particle size like small molecules during the self-assembly process.

For the surface-modified cRGD-PEG-*g*-(PBYP-*ss*-CPT) nanoparticles (abbreviated as cRGD-CPT NPs), Figure 4C,D shows the particle size distribution histogram and corresponding TEM images. After coupling cRGD on the surface of the particles, the *D̅_z_* increased but still range from 50 to 200 nm and the morphology is relatively homogeneous. The particle size distribution in the TEM image is relatively uniform, which is consistent with DLS measurements (size PDI = 0.118). There is a slight difference in the *D̅_z_* measured by DLS (∼152 nm) and TEM (∼120 nm), which is caused by the compression of the dried hydrophilic chains on the nanoparticle surface during the frozen sample preparation.

**Figure 5:**
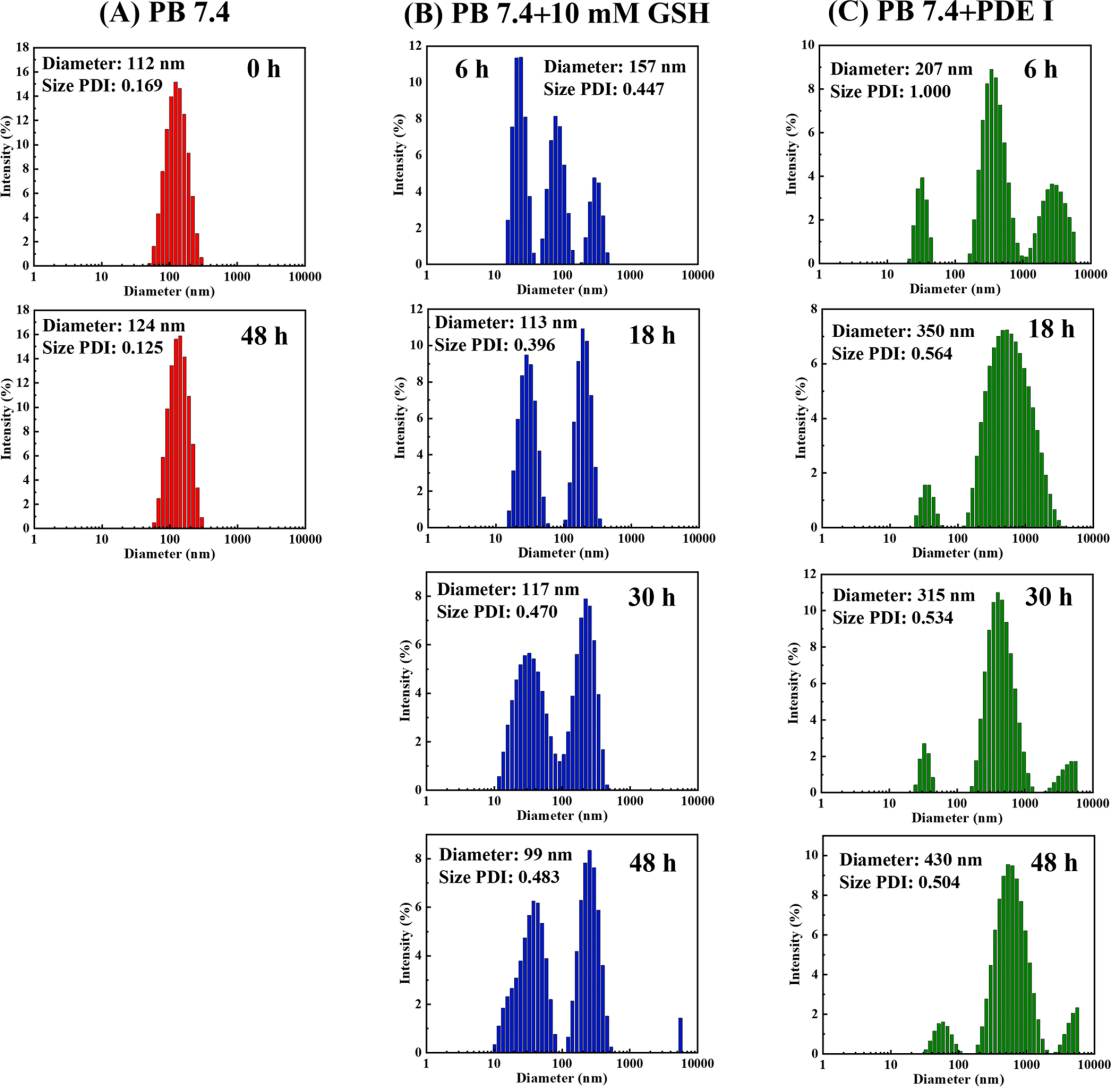
The size distribution histograms of cRGD-CPT NPs under various solutions: (A) PB 7.4, (B) PB 7.4 + 10 mM GSH, and (C) PB 7.4 + PDE I (concentration: 1 mg mL^–1^).

To verify the stability, reduction responsiveness, and enzymatic degradation of the prodrug backbone, the changes in particle sizes under different solutions were investigated. From Figure 5A we can see that the DLS test showed only minor changes in the *D̅_z_* and its distribution after stirring in PB 7.4 buffer for 48 h. However, under the condition of 10 mM GSH, the sizes change significantly and multiple peaks appear, which is due to the breakage of most of the disulfide bonds, causing the destruction of the micelle structure. When PDE I is included, the main chains of polyphosphoester degrade gradually, which results in the breakdown of the amphiphilic polymer structures and the increase of particle sizes. After the drug-loaded nanoparticles were cracked, the hydrophilic and hydrophobic segments were dissolved or randomly reunited respectively, forming irregular particles. Therefore, the histogram of particle size distribution in Figure 5B,C show irregular changes over time.

### *In Vitro* Release of CPT

**Figure 6:**
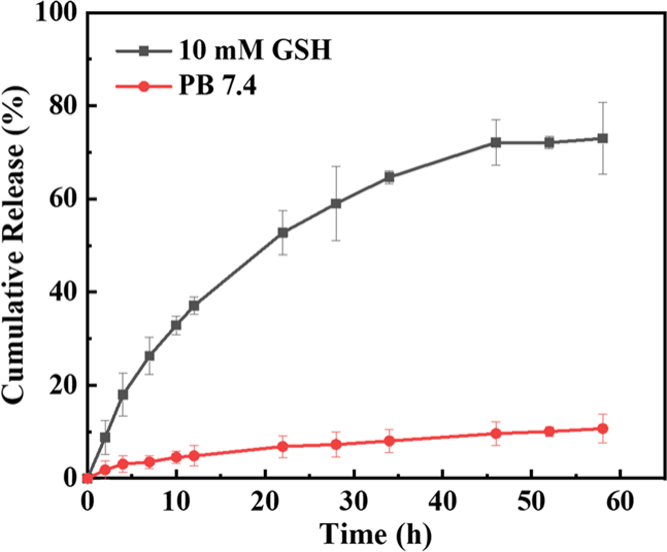
*In vitro* CPT release from cRGD-CPT NPs under different conditions.

For any nanodrug delivery system, the most important factor affecting drug efficacy is whether the nanocarriers can release the original structure of the small-molecule drug in time after accumulation at the lesion. We investigate the reduction-sensitive release by testing the cumulative CPT release under various media. In Figure 6, when cRGD-CPT NPs were placed in 10 mM GSH solution, which mimics the reductive microenvironment of tumor cells, more than 70% of CPT was released within 58 h of dialysis. It is worth noting that the drug was released rapidly in the first 24 h, which facilitates the timely effect of the drug. In contrast, in PB 7.4 buffer simulating a normal cell environment, the CPT leakage rate was approximately 10% at 58 h. This is due to the advantages of chemical bonding of the drug to the carrier: low drug leakage and reduced biological toxicity to normal tissues. The release of drugs from cRGD-CPT NPs is ascribed to the breakage of the disulfide bonds (-*ss*-) under reductive condition. The generation of thiol intermediates that would subsequently undergo intramolecular cyclization, resulting in the native CPT release.^39,43^

### *In Vitro* Hemolysis Activity

**Figure 7:**
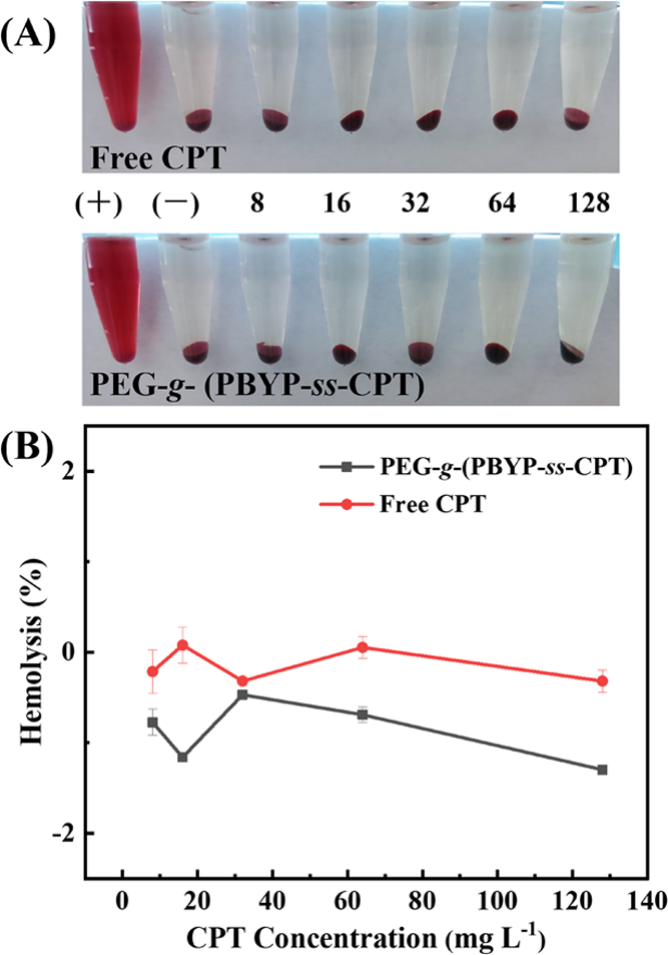
(A) Photographs of free CPT and PEG-*g*-(PBYP-*ss*-CPT) after incubation with erythrocytes for 3 h and (B) percentage of hemolysis [(−): PBS, (+): ultrapure water. The numbers in (A) correspond to the CPT concentration values in (B)].

Some industrial chemicals and organic reagents may cause intolerant high hemolysis (>5%) when combined with red blood cells.^44^ Nanoparticles should not cause breakage of red blood cells during blood circulation. The hemolysis percentage represents the extent to which the erythrocyte cell membrane is disrupted by the material. As shown in Figure 7A, unlike the positive control group, after incubation with erythrocytes at different concentrations, intact erythrocytes settled at the bottom of the centrifuge tube after centrifugation, and no free hemoglobin is observed in the supernatant, indicating that neither free CPT nor PEG-*g*-(PBYP-*ss*-CPT) NPs disrupted the erythrocyte membrane. Consistent with Figure 7A, the hemolysis percentage obtained at CPT concentrations up to 128 mg L^–1^ in Figure 7B is also close to 0, indicating that the NPs have good hemocompatibility.

### *In Vitro* Cytotoxicity

**Figure 8:**
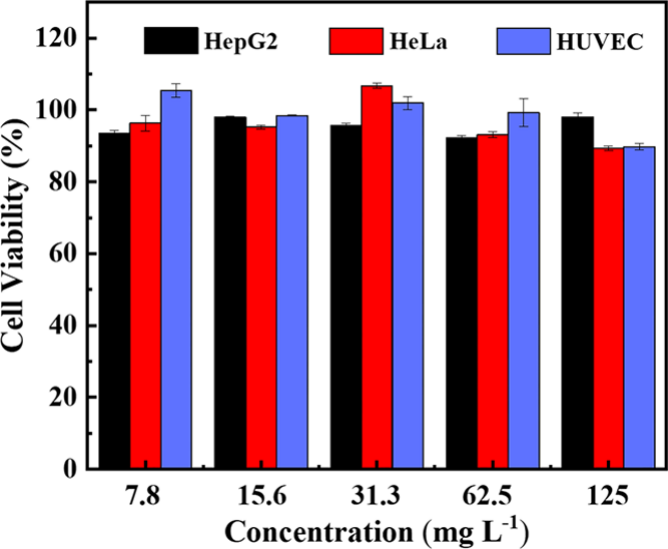
Cell viability of HepG2 cells, HeLa cells, and HUVEC cells incubate with different concentrations of PEG-*g*-PBYP for 48 h.

As we all know, polymer carriers should not cause serious damage to cells of the human body. Biocompatibility is a very important property of polymeric materials used in drug delivery systems. Therefore, we research the *in vitro* cytotoxicity of PEG-*g*-PBYP without CPT against cancer cells and normal cells by MTT assays. Figure 8 shows the cell viability of HepG2 cells, HeLa cells, and HUVEC cells, all of which were incubated with PEG-*g*-PBYP for 48 h. When the PEG-*g*-PBYP concentration was increased to 125 mg L^–1^, there was no decrease in cell survival, indicating that the polymer carrier has no inhibitory effect on both cancer cells and normal cells.

**Figure 9:**
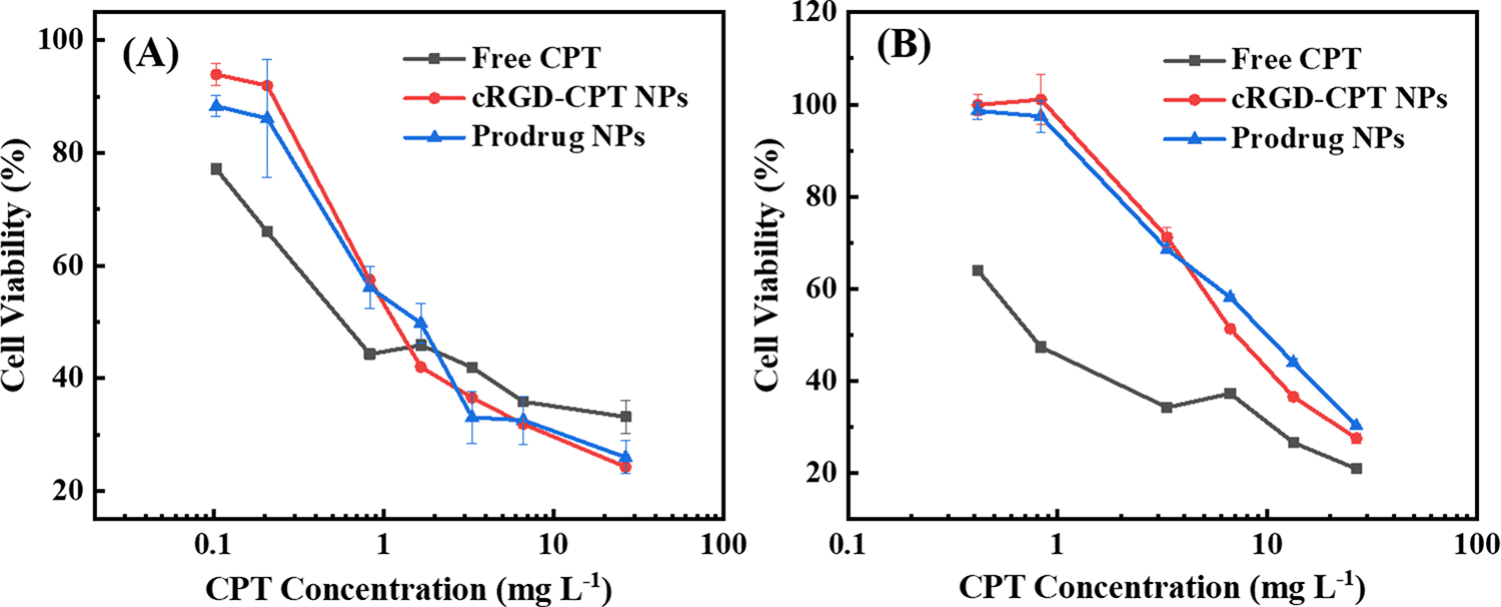
Cell viability of (A) A549 cells and (B) HepG2 cells, incubated with cRGD-CPT NPs, prodrug NPs, and free CPT for 48 h.

Since α_v_β_3_ integrins are overexpressed on the membranes of diverse cancer cell lines, including A549 cells and HepG2 cells, we also investigate the inhibition of cRGD-CPT NPs, prodrug NPs, and free CPT, respectively, against both A549 and HepG2 cells by MTT assay. According to the cell viability versus concentration curves in Figure 9, after 48 h of treatment with different therapeutic agents, the cell viability shows a gradual decrease with increasing concentration of CPT in the therapeutic agent. Moreover, to evaluate the antitumor effect, the half-maximal inhibitory concentration (IC_50_) values are listed in Table 3. The IC_50_ values of free CPT against A549 cells (1.03 mg L^–1^) and HepG2 cells (1.16 mg L^–1^) are separately determined. Compared to free CPT, the prodrug NPs against A549 and HepG2 cells show higher IC_50_ values of 1.93 and 5.94 mg L^–1^, respectively. The cell viability is concentration-dependent but free CPT has the lowest IC_50_ value. It may be due to the biocompatible polyphosphoester reducing the biotoxicity of nanoparticles.^45^ In addition, the cell viability of A549 cells and HepG2 cells coincubation with cRGD-CPT NPs show a regular slow decrease with increasing CPT concentration. However, the concentration dependence of two types of cell coincubation with CPT is completely different. This is due to the defect of the hydrophobic drug, which is promoted by ultrasound to dissolve free CPT in PBS to configure a certain concentration of aqueous solution, but some CPT molecules would aggregate or precipitate, changing the inhibitory effect on tumor cells. Notably, the cRGD-conjugated prodrugs exhibit lower IC_50_ values of 1.36 and 4.35 mg L^–1^ against A549 cells and HepG2, respectively, compared with the prodrug NPs. This suggests that the cRGD-conjugated prodrug has a better tumor-inhibitory effect than the cRGD-free prodrug NPs.

**Table 3:** IC_50_ Values of Free CPT, cRGD-CPT NPs, and Prodrug NPs against A549 Cells and HepG2 Cells, Respectively

IC_50_	A549 (mg L^–1^)	HepG2 (mg L^–1^)
free CPT	1.03	1.16
cRGD-CPT NPs	1.36	4.35
prodrug NPs	1.93	5.94

### Cellular Uptake

**Figure 10:**
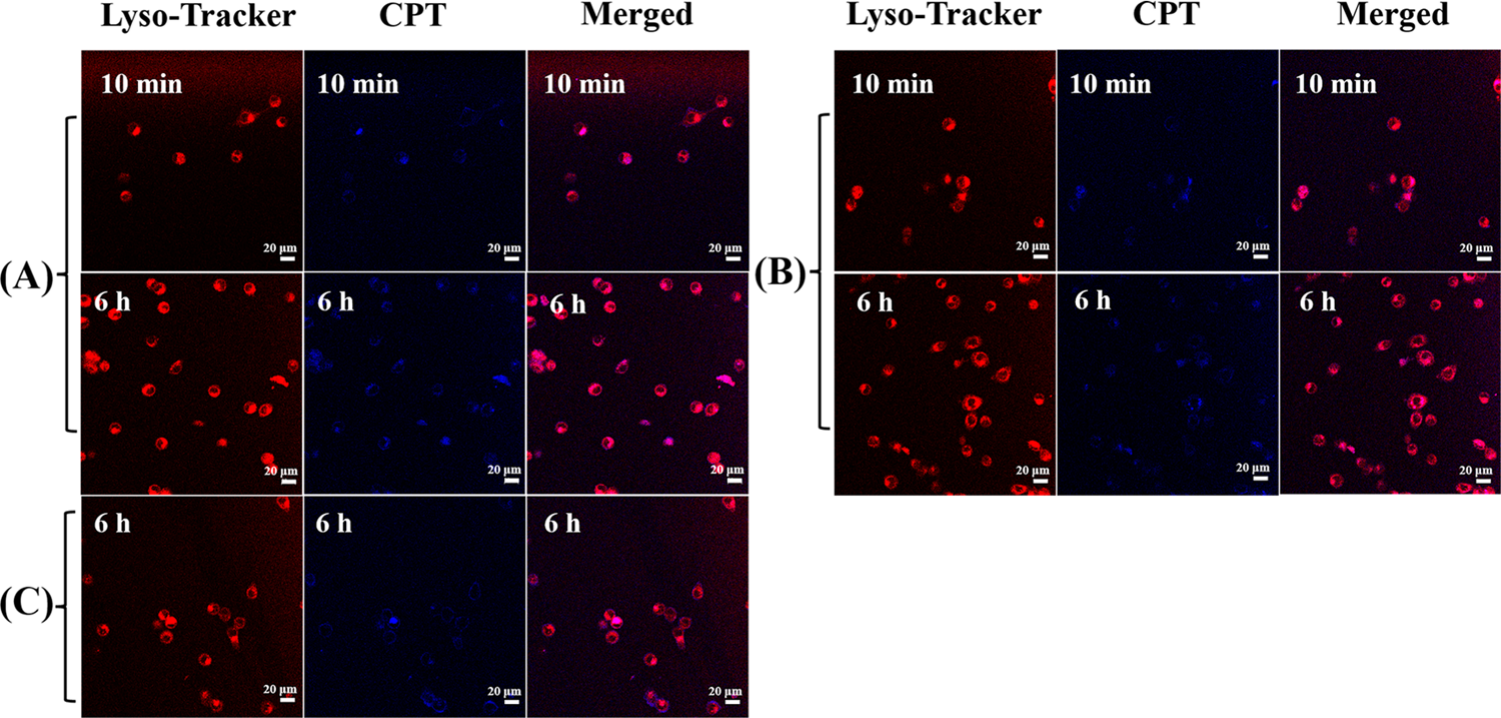
Intracellular fluorescence images of HepG2 cells after treatment with (A) cRGD-CPT NPs, (B) prodrug NPs, and (C) free CPT for 10 min and 6 h. In each row, the three columns from left to right are respectively the fluorescence imaging of stained lysosomes, the fluorescence imaging of CPT in cells, and the combination imaging of two fluorescence (scale bar = 20 μm).

**Figure 11:**
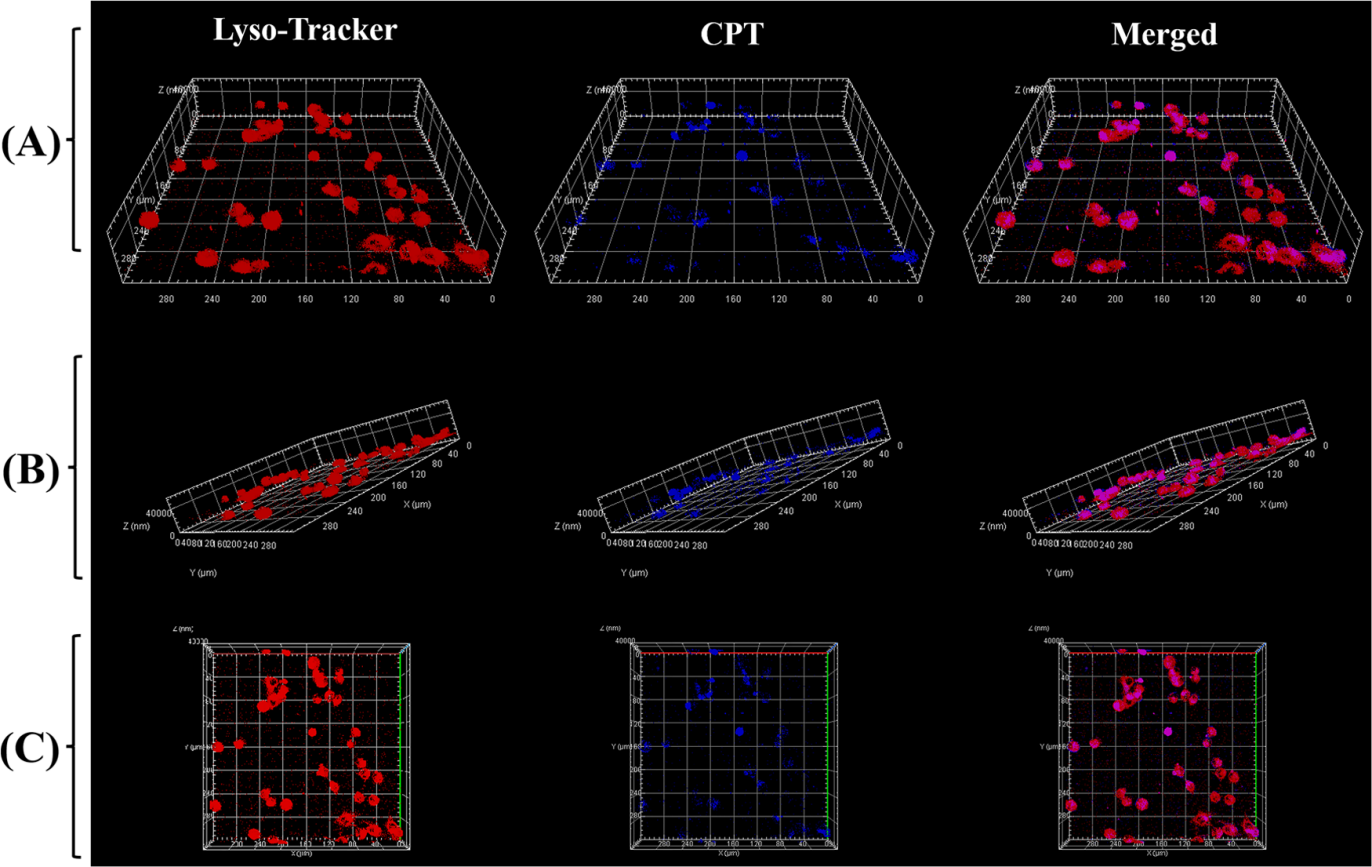
Images taken at different positions of HepG2 cells after 6 h of treatment with cRGD-CPT NPs: (A) front view, (B) side view, and (C) vertical view. The three columns from left to right are respectively the fluorescence imaging of stained lysosomes, the fluorescence imaging of CPT in cells, and the combination imaging of two fluorescence (z-axis length = 40 μm).

The process of drug enrichment in tumor cells was visualized by cellular uptake assay. Figure 10A–C shows the endocytosis of HepG2 cells, incubated with cRGD-CPT NPs, prodrug NPs, and free CPT, respectively. When cRGD-CPT NPs and prodrug NPs were added to confocal dishes for only 10 min, slight CPT fluorescence could be seen in the cells. This is because nanomedicine carriers were rapidly taken up by HepG2 cells. When the incubation time was extended to 6 h, blue fluorescence was significantly enhanced in HepG2 cells incubated with cRGD-CPT NPs. In contrast, the fluorescence intensity of unconjugated cRGD nanoparticle prodrug increased more slowly with the prolongation of the incubation time, and the blue fluorescence was the weakest in HepG2 cells incubated with free CPT. The fluorescence intensity of the cRGD-CPT NPs was significantly the highest among the three medications after 6 h of incubation at the same CPT concentration, which is due to the active targeting effect of cRGD that is more favorable for the endocytosis of the nanoparticles. Meanwhile, we have performed 3D confocal laser scanning microscopy experiments and showed 3D images of the HepG2 cells in different stereo angles. We performed a layer-by-layer scan at this magnification with a total scan depth of 58 μm, (thicker than the thickness of HepG2 cells). Figure 11A–C are the front view, side view, and vertical view 3D images, respectively, where the depth of the z-axis is 40 μm. We can see that the blue fluorescence of CPT is on the same plane as the red fluorescence of lysosomes, confirming that the CPT fluorescence we detected comes from inside the cell, and that nanoparticles are not only adsorbed on the cell surface. The corresponding three-dimensional dynamic video rotated along the x-axis is shown in the Supporting Information.

**Figure 12:**
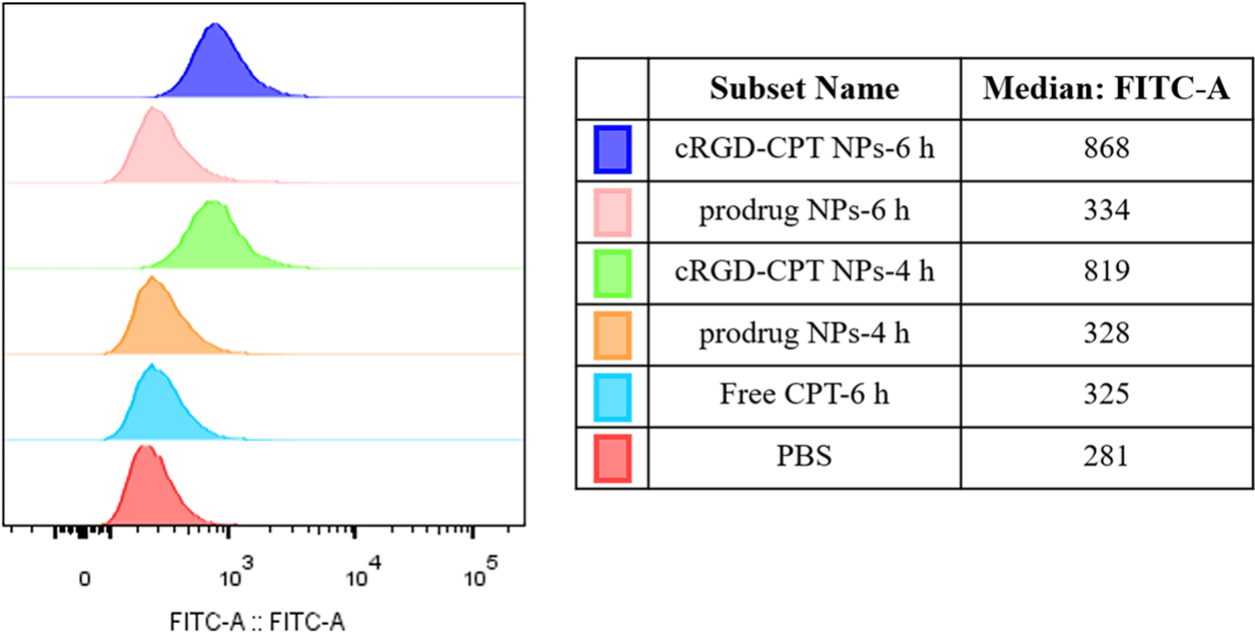
Flow cytometry analysis of HepG2 cells treated with cRGD-CPT NPs, prodrug NPs, and free CPT, respectively. The corresponding mean fluorescence intensity values are shown in the table.

In addition, we also investigated the targeting effect of cRGD-CPT NPs through flow cytometry. Figure 12 shows that compared to the other samples, the CPT fluorescence of HepG2 cells treated with cRGD-conjugated NPs was the strongest and enhanced with time. This can be attributed to the fact that the small molecules are more likely to be excreted outside cells while cRGD-conjugated NPs are actively endocytosed by a receptor-mediated mechanism.^46^ These results suggest that cRGD-CPT NPs have the ability to target HepG2 cells and enhance cellular uptake efficiency.

### *In Vivo* Pharmacokinetics and Biodistribution Study

**Figure 13:**
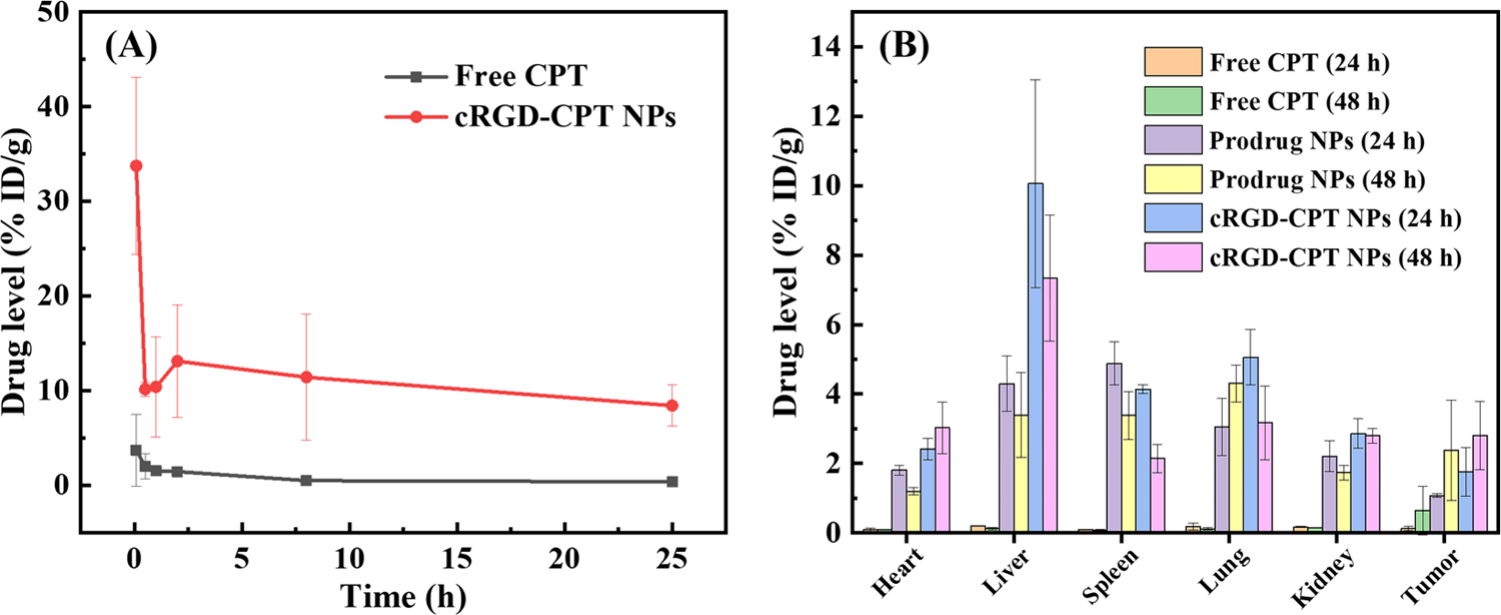
(A) Retention of cRGD-CPT NPs and free CPT in blood after tail intravenous injection over time. (B) Distributions of cRGD-CPT NPs, prodrug NPs, and free CPT in tissues after tail intravenous injection.

The circulation of the drug in the blood of mice can be measured by detecting the concentration of CPT in the blood at different times. Figure 13A shows the retention of cRGD-CPT NPs and free CPT in blood within 24 h of tail vein injection (CPT dose: 5 mg kg^–1^). According to the study, the concentration of free CPT in the blood decreased dramatically and the retention in the body was almost zero after 2 h of injection, as the small-molecule drug can be easily metabolized. In contrast, the circulation time of nanoparticles was significantly longer, and 10% ID g^–1^ remained in the blood 24 h after injection. This demonstrates the advantages of polymeric nanoparticles as drug carriers, that is, reduced adsorption of proteins in the blood and prolonged circulation time.

Based on the long circulation of nanoparticles *in vivo*, we further monitored the distributions of cRGD-CPT NPs, prodrug NPs, and free CPT in tumors, as well as in each tissue. Figure 13B shows that free CPT accumulated at low levels in all tissues, which is due to the rapid metabolism of small-molecule drugs *in vivo*. Benefiting from the EPR effect of the nanodrug delivery system, PEG-*g*-(PBYP-*ss*-CPT) NPs accumulated in tumors and other organs for a longer period of time. In contrast, cRGD-CPT NPs, in addition to being captured by the liver,^3^ accumulated in tumor tissues in higher amounts than PEG-*g*-(PBYP-*ss*-CPT) NPs without the targeting molecule. These results suggest that cRGD-CPT NPs have targeting properties and can improve the drug delivery efficiency. The accumulation of cRGD-CPT NPs (48 h) in tumors was high above the prodrug NPs (48 h), while the accumulation of CPT prodrug NPs (48 h) in other organs was much less than that of cRGD-CPT NPs (48 h). This may be influenced by surface density of PEG, as well as the ratio of PEG to cRGD.^47−49^ Under the condition of continuous administration, the accumulated concentration of CPT in the tumor is enough to inhibit the proliferation of tumor cells, which has a considerable therapeutic effect. The accumulation in the spleen, liver, and lungs may be due to a large number of proteins and inorganic ions in the blood, which have reunited some of the nanoparticles.

## Conclusions

We have used a modified cRGD as a targeting molecule, polyphosphoester as the drug carrier to prepare an active targeting nanodrug and construct a stimuli-responsive cRGD-functional prodrug for precise delivery of antitumor drugs. The polyphosphoester (PBYP) was prepared first by ring-opening polymerization, followed by a one-pot CuAAC “click” reaction between functional camptothecin (CPT-*ss*-N_3_), cRGD-PEG-N_3_, and PBYP. In PB 7.4, the particle sizes of amphiphilic polymeric prodrugs were 127 and 152 nm before and after conjugation with cRGD, respectively. These nanoparticles are stable under physiological conditions, but can efficiently dissociate and release CPT under reducing conditions. At the cellular level, cRGD-CPT NPs are efficiently taken up by tumor cells and exhibited significant effects in inhibiting tumor cell proliferation. The IC_50_ values of cRGD-free prodrug NPs and cRGD-conjugated NPs are 1.93 and 1.36 mg L^–1^ against A549 cells, respectively. Similarly, the IC_50_ value of cRGD-CPT NPs against HepG2 (4.35 mg L^–1^) is also lower than that of cRGD-free prodrug NPs (5.94 mg L^–1^). The increase in the efficiency of tumor cell inhibition is due to the improved recognition capabilities of cRGD. Three-dimensional fluorescence images of HepG2 cells provide ample evidence that CPT is effectively taken up into the cells and not just adsorbed on the cell surface. At the animal level, cRGD-CPT NPs exhibited long-circulation properties, with 10% ID g^–1^ remaining in the bloodstream for 24 hours after injection. In addition, cRGD-CPT NPs showed higher drug accumulation of 2.8% ID g^–1^ in tumor tissues in mice loaded with HepG2 tumors. This work may provide a new way to design and fabricate precisely targeted polymer prodrugs. The obtained amphiphilic polymer prodrug is biocompatible and biodegradable, in which the disulfide bond is easily cleaved in the tumor environment of liver cancer cells, releasing the CPT drug.

## Supporting Information Available

Materials; experimental procedures and all test characterization methods; cell culture; ^1^H NMR spectra of CPT-*ss*-N_3_ and cRGD-PEG-N_3_; GPC curves of PBYP and PEG-*g*-(PBYP-*ss*-CPT); HPLC and UV–vis spectral analyses of the prodrug; and calculation of CAC value of PEG-*g*-(PBYP-*ss*-CPT) (PDF)
Three-dimensional video of intracellular fluorescent image (AVI)

## Supplementary Material

ao2c02683_si_001.pdf

ao2c02683_si_002.avi
