# Supplementary material for: Functional cRGD-Conjugated Polymer Prodrug for Targeted Drug Delivery to Liver Cancer Cells
Source: ACS Omega. 2022 Jun 7;7(24):21325–36. doi: 10.1021/acsomega.2c02683 (PMC9219052; doi:10.1021/acsomega.2c02683)
Supplement: Supplementary file 1 — ao2c02683_si_001.pdf [file ao2c02683_si_001.pdf]

Supporting Information

For

Functional cRGD-Conjugated Polymer Prodrug for Targeted Drug Delivery to Liver Cancer Cells

Ru Zhou[a], Mingzu Zhang[a], Jinlin He[a], Jian Liu[b] Xingwei Sun[c,*], and Peihong Ni[a,*]

[a] College of Chemistry, Chemical Engineering and Materials Science, State and Local Joint Engineering Laboratory for Novel Functional Polymeric Materials, Jiangsu Key Laboratory of Advanced Functional Polymer Design and Application, Suzhou Key Laboratory of Macromolecular Design and Precision Synthesis, Soochow University, Suzhou 215123, P. R. China

[b] Institute of Functional Nano and Soft Materials (FUNSOM), Soochow University, Suzhou, 215123, P. R. China

[c] Intervention Department, The Second Affiliated Hospital of Soochow University, Suzhou 215004, P. R. China

***** To whom correspondence should be addressed. E-mail: phni@suda.edu.cn

Number of pages: 17

Number of figures: 5

Number of tables: 0

Part I. Experimental Section

1. Materials

2,2′-Dithiodiethanol (Sigma-Aldrich), α-bromoisobutyryl bromide (98%, Sigma-Aldrich), sodium azide (NaN_3, Sinopharm Chemical Reagent), camptothecin (CPT, 99%, Beijing Zhongshuo Pharmaceutical Technology Development), *N*,*N*,*N*',*N*'',*N*''-pentamethyl diethylenetriamine (PMDETA, 98%, Sigma-Aldrich), triphosgene (99%, J&K Chemical), NHS-PEG-N_3 ($\bar{M}_n$=2000 g mol^{-1}, Shanghai Ponsure Biotech, Inc.), Cyclo(RGDfK) was synthesized by Shanghai Apeptide Co.,Ltd., L-glutathione (reduced) (GSH, 99%, Shanghai Yuanye Biotechnology), methyl thiazolyl tetrazolium (MTT, 98%, Sigma-Aldrich), Lyso-Tracker Red (Solarbio), and dulbecco's modified eagle medium (DMEM) (HyClone). Triethylamine (TEA, A.R., Enox), dimethyl sulfoxide (DMSO, A.R., Enox) and *N*,*N*-dimethyl-formamide (DMF) were distilled before use. Dichloromethane (CH_2Cl_2, A.R., Enox) was dried over CaH_2 for at least 24 h and distilled before use. Milli-Q water (18.2 MΩ cm at 25 °C) was produced through a water purification system (Simplicity UV, Millipore). 2-(But-3-yn-1-yloxy)-2-oxo-1,3,2-dioxaphospholane (BYP) was prepared and purified according to the literature previously reported.[1,2] All the other chemicals were analytical reagents and used as received unless otherwise mentioned.

2. Synthesis of Reduction-Responsive and Clickable CPT Derivative (CPT-*ss*-N_3)

First, 2,2′-dithiobis[1-(2-bromo-2-methylpropionyloxy) ethane] (HO-*ss*-Br) was

prepared by esterification. A-bromoisobutyryl bromide in anhydrous THF (15 mL) was instilled into the flask under a nitrogen atmosphere, in which 2,2′-dithiodiethanol and TEA were added to THF (40 mL). After that, the solution was further stirred for 12 h at 25 °C. The raw product was concentrated and further purified by silica gel column chromatography using ethyl acetate and CH_2Cl_2 (*v*/*v*: 1/5) as the eluent. Second, the yellow liquid of 2,2′-dithiobis[1-(2-azido-2-methylpropionyloxy) ethane] (HO-*ss*-N_3) was prepared between HO-*ss*-Br and NaN_3. Third, to a 100 mL dry branch flask, CPT (0.84 g, 2.40 mmol), DMAP (0.88 g, 7.21 mg) and triphosgene (0.24 g, 0.81 mmol) in anhydrous CH_2Cl_2 (50 mL) were added and stirred under a nitrogen atmosphere at 25 °C for 1 h. Then, HO-*ss*-N_3 in anhydrous CH_2Cl_2 (15 mL) was instilled into the flask, which was further stirred for 12 h. The mixture was extracted with sodium chloride (NaCl) aqueous solution three times. The organic layer was dried with anhydrous Na_2SO_4. The filtrate was concentrated and further purified by silica gel column chromatography using ethyl acetate as the eluent. Finally, yellow powder was achieved. (CPT-*ss*-N_3, 1.17 g, yield: 79.6%).

3. Synthesis of cRGDfK-modified cRGD-PEG-N_3

The synthesis of cRGD-PEG-N_3 was proceeded by reacting NHS-activated PEG-N_3 chains with amine-terminated cRGDfK.[3] In brief, NHS-PEG-N_3 (80.0 mg, 0.04 mmol) and cRGDfK (24.10 mg, 0.04 mmol) were dissolved in 10 mL of dimethyl sulfoxide (DMSO) into the flask. Then, 4-dimethylaminopyridine (DMAP) (7.33 g, 0.06 mmol) was added and the reaction mixture was stirred at 25 °C under a nitrogen atmosphere for 12 h. The product was dialyzed against deionized water for

48 h and freeze-dried to obtain cRGD-PEG-N_3. (61.0 mg, yield: 58.9%).

4. Synthesis of Polyphosphoester (PBYP)

The polymer PBYP was prepared by ring opening polymerization of 2-(but-3-yn-1-yolxy)-2-oxo-1,3,2-dioxaphospholane (BYP). The reaction was carried out as follows: 8 mL of CH_2Cl_2 was added to a pre-dried 50 mL branched flask under nitrogen. After adding the initiator isopropanol (8.30 mg, 0.14 mmol) and catalyst 1,8-diazabicyclo[5.4.0]undec-7-ene (DBU) (63.0 mg, 0.41 mmol) in turn, the monomer BYP (1.70 g, 9.66 mmol) was added rapidly by differential method using a syringe, and the reaction was carried out under nitrogen protection at 30 °C for 30 min. The mixed product was concentrated and precipitated three times in a mixture of ice anhydrous ether and anhydrous methanol (20:1, *v*/*v*). The precipitated solution was aspirated and the product was dried in a vacuum drying oven for 24 h to obtain pure pale yellow viscous solid PBYP. (1,36 g, yield: 80.1 %).

5. One-Pot Synthesis of cRGD-PEG-*g*-(PBYP-*ss*-CPT) Polymeric Prodrug

The amphiphilic targeted polymer cRGD-PEG-*g*-(PBYP-*ss*-CPT) was synthesised via CuAAC reaction.[4] Briefly, to a Schlenk tube, a mixture of PBYP (51.90 mg, 5.27 μmol), cRGD-PEG-N_3 (17.69 mg, 8.85 μmol) and CPT-*ss*-N_3 (39.40 g，0.06 mmol) was dissolved in 8 mL DMF, and then PMDETA (40.88 mg, 0.24 mmol) was added to the mixture before freezing vacuum for 3 min. After the mixture thawed, CuBr (16.91 mg, 0.12 mmol,) was also added to the Schlenk tube. The reaction mixture was stirred magnetically at 35 °C under nitrogen. After 12 h of reaction, the

solution was terminated by quickly cooling the Schlenk tube in an ice-water bath, followed by dialysis (MWCO 3500) against DMF and deionized water for 48 h to remove copper ions. The solution was collected and then freeze-dried under vacuum to obtain the product cRGD-PEG-*g*-(PBYP-*ss*-CPT). (57.2 mg, yield: 54.9%).

6. Characterization

^{1}H NMR spectra were recorded on a 300 MHz spectrometer (INOVA-300, Varian), using $CDCl_3$ or D_2O as the solvents and tetramethylsilane (TMS) as the internal standard. The number-average molecular weights ($\bar{M}_n$) and dispersity (*Đ*) of PBYP and PEG-*g*-(PBYP-*ss*-CPT) were analyzed by gel permeation chromatography (GPC) instrument (HLC-8320, Tosoh) using polystyrene as the standard and DMF including 0.1 wt% LiBr as the eluent. The ultraviolet-visible (UV-vis) absorption spectra were conducted on a UV-vis spectrophotometer (UV-vis 1900, Shimadzu), and fluorescence spectra were recorded on a fluorescence spectrophotometer (Cary Eclipse, Agilent). The self-assembly behavior of the polymer nanoparticles and morphological changes under different conditions were explored by DLS and TEM. Various methods were used to analyze the *in vitro* and *in vivo* effects of nanoparticles.

7. MALDI-TOF MS Measurement

Matrix-assisted laser desorption/ionization time-of-flight (MALDI-TOF) mass spectra were recorded on an AXIMA Performance-MALDI TOF/TOF mass spectrometer (Shimadzu Biotech Manchester, UK) equipped with a N_2 laser emitting at 337 nm. All spectra were measured in the linear mode. The optimized mass spectra

were obtained with anthratriol as the matrix. Typically, the samples were dissolved in a 5 mg mL^{-1} matrix with 50% acetonitrile-0.1% trifluoroacetic acid (TFA) and crystallized in a MALDI target. The typical spot size on the standard 384 spot stainless-steel targets was about 2 mm. 250-500 laser shots were acquired for each mass spectrum, and the spectra were obtained at a laser power that was optimized to maximize resolution and peak intensity.

8. Self-assembly Behavior

The critical aggregation concentration (CAC) of PEG-*g*-(PBYP-*ss*-CPT) nanoparticles was measured by the fluorescence probe method using pyrene as the fluorescence probe. Briefly, to group vials 50 μL of pyrene solution in acetone (6×10^{-7} M) was added and acetone was removed under vacuum conditions. Then 5 mL polymer aqueous solutions of different concentrations were added, followed by ultrasonication for 30 min and stirring for 48 h at room temperature. A fluorescence spectrophotometer was used to analyze the intensity of pyrene. The excitation was set at 335 nm, while emission spectra were recorded with a 5 nm slit width over a wavelength from 350 to 550 nm. The intensity ratio (I_{383}/I_{372}) of the peak (383 nm, I_{383}) to the peak (372 nm, I_{372}) from the emission spectra was analyzed as a function of the logarithmic concentrations of polymeric prodrug. The intersection was determined as the CAC value.

9. *In vitro* Enzymatic Degradation

The degradation of polymers as drug carriers is an indispensable property. To

verify the enzymatic degradation performance of the polyphosphoester backbone, ^{1}H NMR was used to test the degradation products at different times in the presence of phosphodiesterase I (PDE I). In detail, 30 mg of PEG-*g*-PBYP was dissolved in 15 mL of buffer solution containing 0.5 mg mL^{-1} phosphodiesterase I (PDE I) and 5 mg mL^{-1} $MgCl_2·6H_2O$, which was then divided into three portions and immersed in a thermostatic shaker at 37 °C. Each portion was freeze-dried at a predetermined time point and then subjected to ^{1}H NMR analysis.

10. *In vitro* CPT Release from cRGD-PEG-*g*-(PBYP-*ss*-CPT)

The *in vitro* CPT release behavior of cRGD-CPT NPs was studied in the following process. First, 15 mg of cRGD-PEG-*g*-(PBYP-*ss*-CPT) prodrug was ultrasonically dissolved in 30 mL phosphate buffer solution (0.1 M, PB 7.4) and stirred for 4 h. Second, each 2.5 mL of the prodrug micellar solution was transferred into a dialysis membrane (MWCO 3500), and then the dialysis membranes were placed into a series of tubes with 30 mL of two different buffer solutions, which were phosphate buffer solution (0.1 M, pH 7.4) with 0.5 wt% tween 80, phosphate buffer solution (0.1 M, pH 7.4) with 0.5 wt.% tween 80 and 10 mM GSH. These tubes were placed into a constant temperature shaker at 37 °C. At predetermined intervals, 5 mL of the solution was taken out and replenished with an equal volume of the corresponding fresh buffer solution. Fluorescence measurements were carried out to determine the content of the released CPT. The excitation was set at 365 nm while emission spectra were recorded with a 5 nm slit width over a wavelength from 390 to 550 nm.

11. Cell Culture

Human umbilical vein endothelial cells (HUVEC cells), human hepatocellular carcinoma cells (HepG2 cells), human non-samll cell lung carcinoma cells (A549 cells) and human cervical cancer cells (HeLa cells) were obtained from American Type Culture Collection (ATCC) and cultured in high glucose DMEM containing 10% fetal bovine serum (FBS) and 1% penicillin/streptomycin solution. Both cell lines were passaged once every 2 days and incubated at 37 °C in an atmosphere containing 5% CO_2 and certain humidity.

12. Animals

Female BALB/c mice (5 weeks old, 18-22 g) were purchased from Hangzhou Ziyuan Laboratory Animal Technology Co., Ltd. Animals were housed according to AAALAC (Association for Assessment and Accreditation of Laboratory Animal Care) guidelines. All animal-related experiments were performed in full compliance with institutional guidelines and were approved by the The Second Affiliated Hospital of Soochow University Administrative Advisory Committee on Animal Use and Care.

13. *In Vitro* Hemolysis Activity

The blood compatibility of free CPT and PEG-*g*-(PBYP-*ss*-CPT) was evaluated by spectrophotometry technique according to previous research.[5,6] The Second Affiliated Hospital of Soochow University offered the mouse blood. Typically, physiological saline (2 mL) was added into blood sample (1 mL). After gentle blowing with pipettes, red blood cells (RBCs) were isolated from serum by

centrifugation at 500 g for 10 min. The RBCs were further washed three times with PBS and then diluted to 10 mL in PBS. Then, diluted RBC suspension (0.5 mL) was added into 0.5 mL of free CPT and PEG-*g*-(PBYP-*ss*-CPT) at different concentrations in PBS and mixed by vortexing gently. The final CPT concentration ranges from 8 to 128 mg L^{-1}. Herein, RBCs incubated with PBS (−) and Milli-Q water (+) were used as the negative and positive controls, respectively. All the samples were placed on a rocking shaker in an incubator at 37 °C for 3 h. After that, the samples were centrifuged at 10000 rpm for 5 min and the supernatant (100 μL) of all the samples were transferred into a 96-well plate. The hemoglobin absorbance in the supernatant was measured at 540 nm, by a microplate reader (Bio Rad 680) to obtain the optical density (OD) values. The percent hemolysis value was calculated according to eq (1):

$$\text{Hemolysis (\%)} = \frac{\text{OD}_{\text{sample}} - \text{OD}_{\text{negative control}}}{\text{OD}_{\text{positive control}} - \text{OD}_{\text{negative control}}} \times 100 \qquad (1)$$

where $\text{OD}_{\text{sample}}$, $\text{OD}_{\text{negative treated}}$ and $\text{OD}_{\text{positive control}}$ represent the OD values of the wells treated with samples, the negative control wells with PBS and the positive control wells with Milli-Q water, respectively. The data are gathered and processed as the average values with standard deviations.

14. *In vitro* Cytotoxicity Test

A methyl thiazolyl tetrazolium (MTT) assay was used to evaluate the cytotoxicity of the PEG-*g*-PBYP, free CPT, PEG-*g*-(PBYP-*ss*-CPT) (abbreviated to prodrug NPs) and cRGD-PEG-*g*-(PBYP-*ss*-CPT) (abbreviated to cRGD-CPT NPs). Cells were seeded in 96-well plates at a density of 5×10^4 cells per well in 100 μL of

high glucose DMEM medium, and incubated at 37 °C in a 5% CO_2 atmosphere for 12 h. Then, 25 μL of the sample solution, at different concentrations, were separately added into each well. After incubation for 48 h, 25 μL of MTT solution (5 mg mL^{-1} in PBS) was added to each well. Subsequently, the cells were incubated at 37 °C for an additional 4 h allowing the viable cells to induce MTT into purple formazan crystals. DMEM medium was removed and 150 μL of DMSO was added to each well. Finally, the optical density (OD) was measured on a microplate reader (Bio Rad 680, U.S.A.) at 570 nm. The cell viability was calculated by eq (2):

$$\text{Cell viability (\%)} = \frac{OD_{treated}}{OD_{control}} \times 100 \qquad (2)$$

where $OD_{treated}$ and $OD_{control}$ represent the OD values of the treated wells in the presence of samples and the control wells in the absence of samples. The data are presented as the average values with standard deviations.

15. Cellular Uptake of cRGD-PEG-*g*-(PBYP-*ss*-CPT) Micelles

The cellular uptake and intracellular release behaviors of free CPT and CPT prodrug in HepG2 cells were investigated by the confocal laser scanning microscope (Zeiss, LSM 800). Typically, HepG2 cells were seeded in a Φ=20 mm confocal dish at a density of 2×10^5 cells per well and cultured in high glucose DMEM culture medium at 37 °C under a 5% CO_2 atmosphere for different times. Afterward, the culture medium was removed. The cells were washed with PBS three times and stained with Lyso-Tracker Red (0.1 μL mL^{-1}) for 1 h, followed by washing with PBS three times. The culture medium was removed within the designed time, and fresh

culture medium containing sample was added for further culture. The concentration of CPT in all newly added media was 13 mg L^{-1}. The images were then captured at excitation wavelengths of 589 nm (red) and 363 nm (blue).

16. Flow cytometry

HepG2 cells were inoculated at a density of 2×10^5 cells cm^{-2} in 6-well plates and incubated in 2 mL of DMEM containing 10% FBS for 12 h. Subsequently, the old medium was removed at different time points and DMEM containing 10% sample was added and incubated for 4 h and 6 h, respectively. After removing the medium and subsequently washing with PBS solution thrice, the cells were collected for flow cytometry quantitative analysis (BD FACSCalibur, USA) with an excitation at 365 nm.

17. Pharmacokinetics and *In vivo* Biodistribution of Nanoparticles

Pharmacokinetics and *in vivo* biodistribution of nanoparticles studies were performed according to the previous reports.[7] Nanoparticles with diameters between 50 and 200 nm usually show a longer retention time in the circulation compared to small molecule drugs.[8] Therefore, pharmacokinetic studies were performed by tail vein injection of free CPT and cRGD-CPT NPs solution (5 mg kg^{-1} CPT) in BALB/c mice (18-22 g). Blood was collected from the tail vein for 0 min before administration (blank group), and then for 5 min, 30 min, 1 h, 2 h, 4 h, 8 h, and 24 h after administration, with a volume of approximately 10 μL each time, and the blood was placed in a weighed centrifuge tube containing 20 μL of sodium heparin. The blood

was weighed again and the blood mass was calculated. Each centrifuge tube was added with 1 mL of extraction solution, sonicated for 5 min, and left overnight at -20 °C. The extraction solution was 1 mL of methanol with 50 μL of aqueous GSH, which can induce disulfide bond breakage in cRGD-CPT NPs. Finally, centrifuge tubes were centrifuged at 12000 rpm at 4 °C. The supernatant was taken in a 96-well plate black plate and tested with an enzyme marker at an excitation wavelength of 365 nm and an emission wavelength of 488 nm. The distribution of the drug in the blood was calculated as follows eq (3):

$$\text{Drug level } (\%\text{ID / g}) = \frac{I_x - I_0}{m \times (I_s \times V)} \times 100 \qquad (3)$$

where I_x is the fluorescence intensity of the extract at different blood sampling times, I_0 is the fluorescence intensity of the blood extract before administration, m is the mass of blood (g), I_s is the fluorescence intensity of the injected sample diluted 1000 times, and V is the single injection volume (μL). The blood extraction time was used as the horizontal coordinate and the drug level (%ID/g) was used as the vertical coordinate to make a dotted line graph of the blood circulation of the drug in mice.

Eighteen mice were selected and divided into 3 groups of 3 mice each, and the mice were injected with free CPT, prodrug NPs and cRGD-CPT NPs in the tail vein at a dose of 5 mg kg^{-1}. The mice were sacrificed at 24 h and 48 h, respectively, and the following organs were removed: heart, liver, spleen, lung kidney and tumor. The surfaces were washed with PBS. Place them in centrifuge tubes and weigh them. After grinding with a homogenizer, 1 mL of extraction solution was added to each tube,

sonicated for 5 min and left overnight at -20 °C. Finally, the tubes were centrifuged at 12000 rpm at 4 °C. The supernatant was extracted in a 96-well black plate and tested with an enzyme marker at an excitation wavelength of 365 nm and an emission wavelength of 488 nm. The distribution of the drug in tissues was expressed as a percentage of the total injected drug per gram of tissue (%ID/g), and the test results were calculated and analyzed according to eq (4)：

$$\text{Drug level (\%ID / g)} = \frac{I_x}{m \times (I_s \times V)} \times 100 \qquad (4)$$

where I_x is the fluorescence intensity of extracts from different tissues, m is the blood mass (g), I_s is the fluorescence intensity of injected sample solution diluted 1000 times, and V is the single injection volume (μL). The distribution of the drug in each tissue in mice is shown as a histogram with the organ name as the horizontal coordinate and the drug level (%ID/g) as the vertical coordinate.

Part II. Supplementary Figures

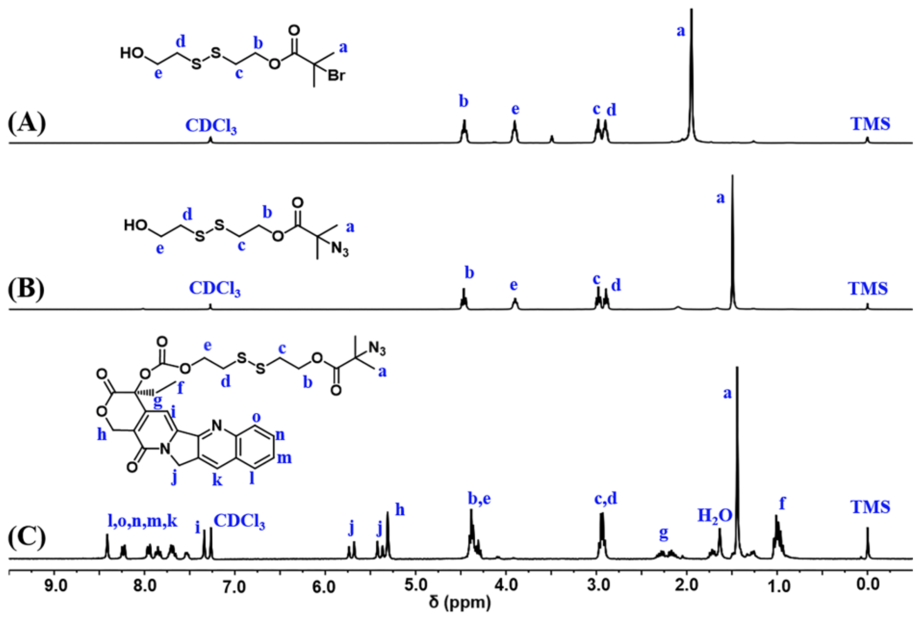

Figure S1. ^{1}H NMR spectra of (A) HO-*ss*-Br, (B) HO-*ss*-N_3 and (C) CPT-*ss*-N_3 in $CDCl_3$.

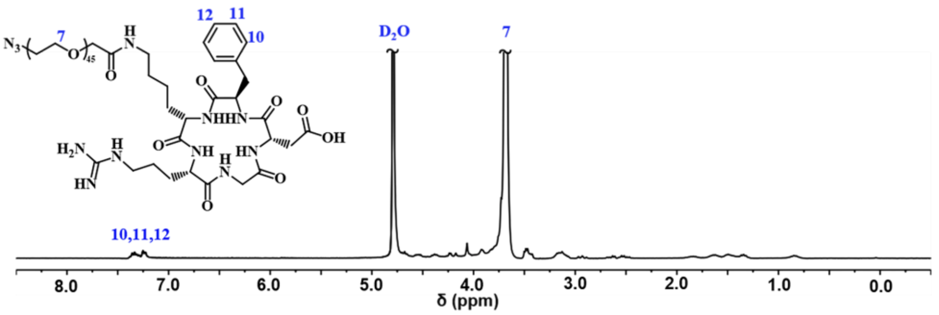

Figure S2. ^{1}H NMR spectrum of cRGD-PEG-N_3 in D_2O.

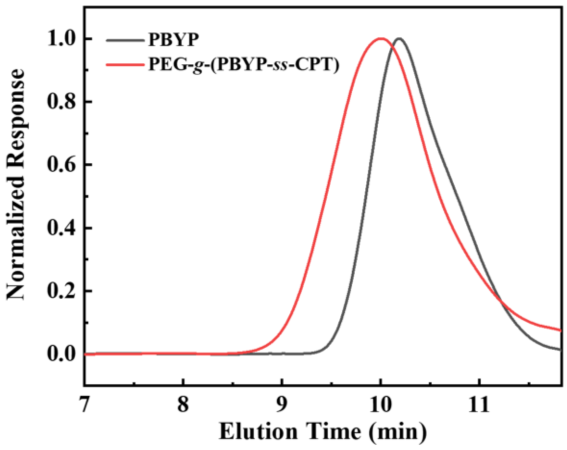

Figure S3. GPC traces of PBYP and PEG-*g*-(PBYP-*ss*-CPT) (eluent: DMF).

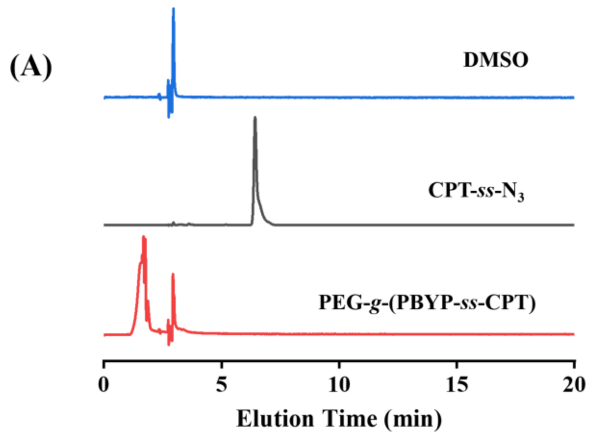

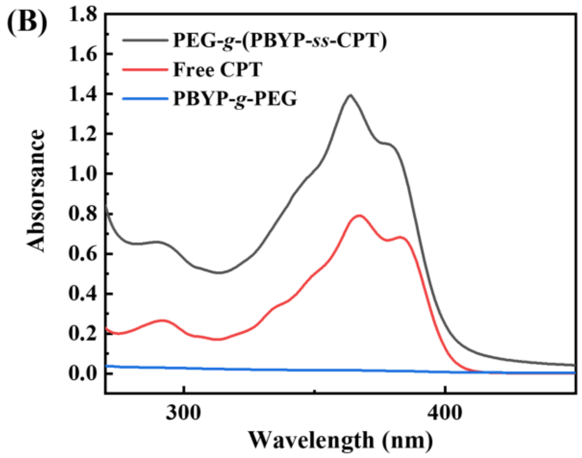

Figure S4. (A) HPLC analyses results of CPT-*ss*-N_3 and PEG-*g*-(PBYP-*ss*-CPT). HPLC analyses were performed with acetonitrile-water (75/25, *v*/*v*) as the mobile phase at 30 °C with a flow rate of 1.0 mL min^{-1}. (B) UV-vis spectra of PEG-*g*-(PBYP-*ss*-CPT), free CPT and PEG-*g*-PBYP.

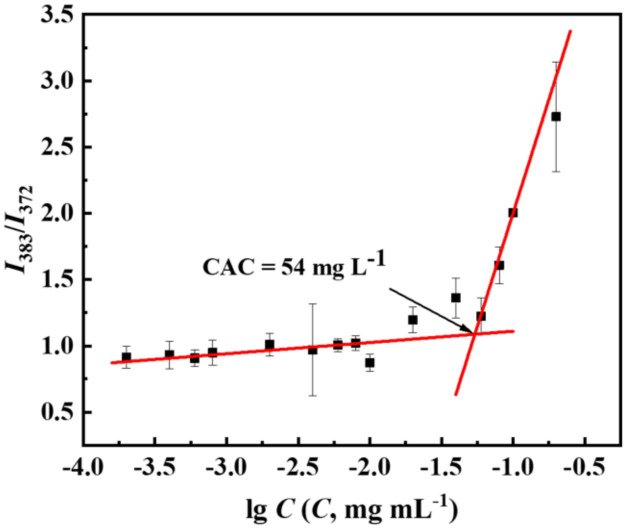

Figure S5. Intensity ratios (I_{383}/I_{372}) as a function of logarithm concentration of PEG-*g*-(PBYP-*ss*-CPT) in aqueous solution.

REFERENCES

1. Zhang, S.; Li, A.; Zou, J.; Lin, L. Y.; Wooley, K. L. Facile Synthesis of Clickable, Water-Soluble and Degradable Polyphosphoesters. *ACS Macro. Lett.* **2012,** *1*, 328-333. DOI: 10.1021/mz200226m.
2. Steinbach, T.; Schröder, R.; Ritz, S.; Wurm, F. R. Microstructure Analysis of Biocompatible Phosphoester Copolymers. *Polym. Chem.* **2013,** *4*, 4469-4479. DOI: 10.1039/c3py00563a.
3. Kasper, M. A.; Gerlach, M.; Schneider, A. F. L.; Groneberg, C.; Ochtrop, P.; Boldt, S.; Schumacher, D.; Helma, J.; Leonhardt, H.; Christmann, M.; Hackenberger, C. P. R. N-Hydroxysuccinimide-Modified Ethynylphosphonamidates Enable the Synthesis of Configurationally Defined Protein Conjugates. *Chembiochem* **2020,** *21*, 113-119. DOI: 10.1002/cbic.201900587.
4. Yan, K. C.; Sedgwick, A. C.; Zang, Y.; Chen, G. R.; He, X. P.; Li, J.; Yoon, J.; James, T. D. Sensors, Imaging Agents, and Theranostics to Help Understand and Treat Reactive Oxygen Species Related Diseases. *Small Methods* **2019,** *3*, 1900013-1900054. DOI: 10.1002/smtd.201900013.
5. Liu, K. F.; Zheng, D.; Zhao, J. Y.; Tao, Y. H.; Wang, Y. S.; He, J.; Lei, J. D.; Xi, X. J. pH-Sensitive Nanogels Based on the Electrostatic Self-Assembly of Radionuclide ^{131}I Labeled Albumin and Carboxymethyl Cellulose for Synergistic Combined Chemo-Radioisotope Therapy of Cancer. *J. Mater. Chem. B* **2018,** *6*, 4738-4746. DOI: 10.1039/c8tb01295d.

6. Zohreh, N.; Rastegaran, Z.; Hosseini, S. H.; Akhlaghi, M.; Istrate, C.; Busuioc, C. pH-Triggered Intracellular Release of Doxorubicin by a Poly(Glycidyl Methacrylate)-Based Double-Shell Magnetic Nanocarrier. *Mater. Sci. Eng. C* **2021,** *118*, 111498-111508. DOI: 10.1016/j.msec.2020.111498.
7. Sindhwani, S.; Syed, A. M.; Ngai, J.; Kingston, B. R.; Maiorino, L.; Rothschild, J.; MacMillan, P.; Zhang, Y. W.; Rajesh, N. U.; Hoang, T.; Wu, J. L. Y.; Wilhelm, S.; Zilman, A.; Gadde, S.; Sulaiman, A.; Ouyang, B.; Lin, Z.; Wang, L. S.; Egeblad, M.; Chan, W. C. W. The Entry of Nanoparticles into Solid Tumours. *Nat. Mater.* **2020,** *19*, 566-575. DOI: 10.1038/s41563-019-0566-2.
8. Blanco, E.; Shen, H.; Ferrari, M. Principles of Nanoparticle Design for Overcoming Biological Barriers to Drug Delivery. *Nat. Biotechnol.* **2015,** *33*, 941-951. DOI: 10.1038/nbt.3330.
